# The Crucial Role of Solvation Forces in the Steric Stabilization of Nanoplatelets

**DOI:** 10.1021/acs.nanolett.2c02848

**Published:** 2022-12-09

**Authors:** Nanning Petersen, Martin Girard, Andreas Riedinger, Omar Valsson

**Affiliations:** † Max Planck Institute for Polymer Research, Mainz D-55128, Germany; ‡ Department of Chemistry, University of North Texas, Denton, Texas 76201, United States

**Keywords:** nanoparticles, nanoplatelets, quantum wells, interaction forces, solvation forces, steric stability, molecular dynamics simulations

## Abstract

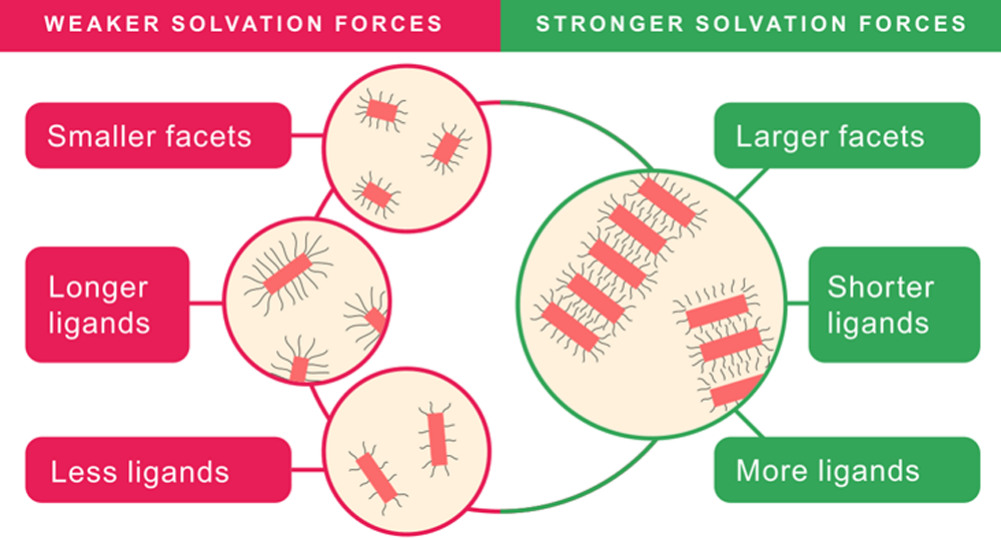

The steric stability of inorganic colloidal particles in an apolar solvent is usually described in terms of the balance between three contributions: the van der Waals attraction, the free energy of mixing, and the ligand compression. However, in the case of nanoparticles, the discrete nature of the ligand shell and the solvent has to be taken into account. Cadmium selenide nanoplatelets are a special case. They combine a weak van der Waals attraction and a large facet to particle size ratio. We use coarse grained molecular dynamics simulations of nanoplatelets in octane to demonstrate that solvation forces are strong enough to induce the formation of nanoplatelet stacks and by that have a crucial impact on the steric stability. In particular, we demonstrate that for sufficiently large nanoplatelets, solvation forces are proportional to the interacting facet area, and their strength is intrinsically tied to the softness of the ligand shell.

Stabilization of colloidal solutions generally relies on a fine balance between different forces. In aqueous solutions, the interaction between charged particles can be described by the DLVO (Derjaguin, Landau, Verwey, Overbeek) theory. Based on this theory, the stability of the colloidal solution results from a balance between the attractive van der Waals forces and the repulsive electrostatic forces.^1^

In apolar solvents, where electrostatic interactions are negligible, colloid solutions are typically sterically stabilized. This is done by covering colloid particle surfaces with ligands. In this environment, usually three effects are taken into account to describe the stability: the attraction due to the van der Waals interaction between the particle crystals, the free energy of mixing of the ligands with the solvent, and the compression of the ligands.^2,3^

In the case of nanoparticles, especially in the case of weak van der Waals interactions between the nanocrystals, these simplified models are not sufficient. Here the discrete nature of the ligand and solvent molecules have to be taken into account.^4^ The discrete nature of the solvent can cause a restructuring of the fluid around colloidal nanoparticles.^5,6^ When two nanoparticles come close to each other, this effect can intensify, and the solvent can form layers. Due to this layering and due to changes in the entropy of the solvent, solvation forces can emerge and become prominent.^1,7−10^ These forces are also referred to as structural forces or, in the case of an aqueous environment, hydration forces.^1,10^

While various studies have shown the importance of hydration forces for the interaction of nanoparticles with hard and smooth surfaces in aqueous environments,^11−13^ there is a knowledge gap for solvation forces between ligand coated nanoparticles in apolar solutions.

Studies in which the solvent is explicitly presented can offer an insight into the issue. While for spherical ligand coated nanoparticles solvation forces seem to play a minor role,^14−16^ for ligand coated nanoparticles with extended flat surfaces, this is not necessarily the case. For example, Widmer-Cooper et al. have shown that solvent layering plays an important role in the interaction of nanorods with larger facets.^17,18^ We can explain this behavior by the properties of the ligand shell. The density of a ligand shell on a curved surface decreases rapidly toward the outside, while it decreases only slowly on a flat one.^19^ Since layer formation of the solvent depends on the softness and the uniformity of the surface,^1^ it is more pronounced between the facets of the nanorods than between the curved surfaces of the spherical nanoparticles.

Jana et al. have studied the steric stability and precipitation dynamics of ligand coated CdSe nanoplatelets in apolar alkane solvent.^20^ The precipitation of colloidal particles usually begins with the aggregation of individual particles into larger objects.^2^ In the case of nanoplatelets, they show the tendency to self-assemble into stacks. Thereby, the stack formation and precipitation kinetics depend on parameters like the concentration of the platelets, the ligand length, and the type of the ligands.^20^

At first glance, the experimentally observed behavior seems to be in good agreement with the model of a balance between the van der Waals attraction, the free energy of mixing, and the ligand compression. For example, Jana et al. observed a higher colloidal stability with longer ligands and a faster precipitation with shorter ligands.^20^ However, a closer look reveals that the van der Waals attraction between the CdSe nanocrystals is too weak to be the cause of stack formation and precipitation (≤ 1 *k*_B_*T*, see Section S1.1 in the Supporting Information (SI) for further discussion).^1,21−23^ Therefore, another type of interaction has to be responsible for the strong attraction between the nanoplatelets.

Nanoplatelets show characteristics that favor solvation forces. Among the different shapes of nanoparticles, platelets have extraordinarily large facets in relation to their particle size.^24^ Additionally, CdSe nanoplatelets are synthesized typically with very dense ligand shells.^19,25−28^

Here, we address the question of whether solvation forces can explain the observed stack formation and precipitation kinetics. We study the interaction of nanoplatelets in octane solvent using coarse grained molecular dynamics simulations. Such a coarse grained approach is well suited for identifying qualitative trends, while at the same time, the computational effort is reduced due to the decreased number of particles in the simulation.

Our model system can be seen in Figure 1. We model the nanoplatelets by surface beads. We consider platelets with base facet areas between 2.2 and 55.7 nm^2^ (see Tables S5–S13 in the SI). Unless otherwise specified, the nanoplatelet thickness is 1.5 nm. Ligand and solvent molecules and their interactions are described by the MARTINI force field,^29^ in which each bead generally represents four heavy atoms such as C, N, or O. This force field has been used to investigate similar nanoparticle systems.^30−33^ In addition, we show in Section S1.2 of the SI that the MARTINI force field and a united atom simulation setup^34,35^ result in a similar solvent layer formation at the ligand-solvent interface.

**Figure 1:**
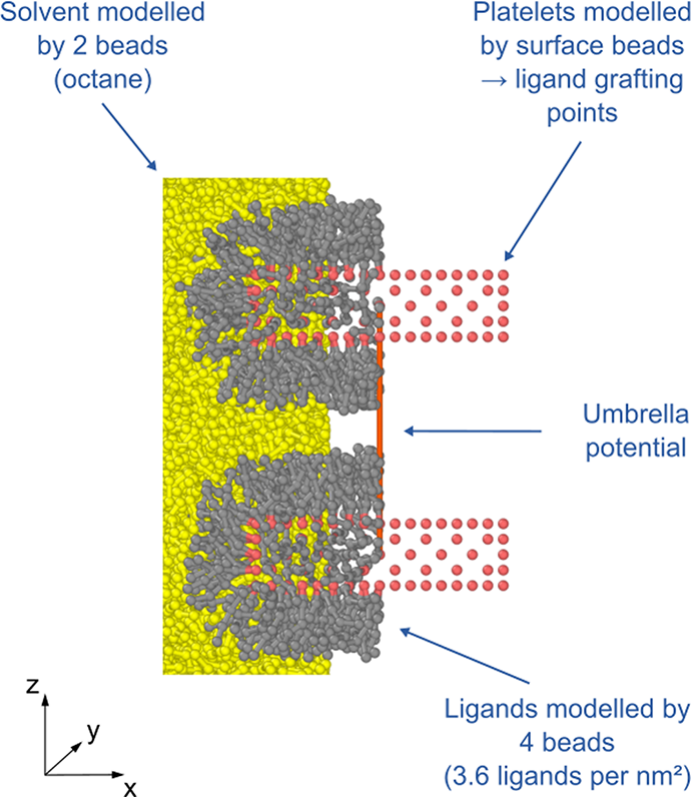
Model system setup. CdSe beads are shown in red, ligand beads in gray, and solvent beads in yellow. The MARTINI force field is used. The length of ligand molecules and the ligand grafting density are varied as well as the nanoplatelet size. In umbrella sampling simulations, the movement of the nanoplatelets is constrained to the *z*-axis.

If not otherwise stated, we model the ligands by four MARTINI C1 beads. This corresponds to an aliphatic lipid tail with a length of 16 carbon atoms. The surface beads are used as grafting points. Unless otherwise specified, a ligand grafting density of 3.6 ligands per nm^2^ is used. The octane solvent molecules are modeled by two MARTINI C1 beads.

To run our molecular dynamic simulations, we use the HOOMD package (v2.6.0).^36−38^ Our simulations are performed at a constant temperature of 300 K and a constant pressure of 1 atm (NPT) in cubic, periodic simulation boxes. For the barostat and thermostat, we employ the standard HOOMD NPT integrator, based on the Martyna-Tobias-Klein equations of motion.^39−41^ To simplify the system setup, we utilize the HOOBAS molecular builder tool.^42^ We use OVITO^43^ to analyze the simulations and take snapshot images. Complete simulation details as well as tables containing facet areas, numbers of solvent molecules, and simulation box sizes can be found in the SI.

We start by investigating the stability of nanoplatelet stacks by performing unconstrained molecular dynamics simulations on preformed stacks. Here, we consider platelets with base facet areas between 20.0 and 55.7 nm^2^ (see Tables S5 and S6 in the SI). We consider stacks consisting of two or three nanoplatelets that are relaxed to the global minimum in the face to face orientation that is taken along the *z* direction (later called first minimum, vide infra). We characterize the behavior of these nanoplatelets over a simulation time of 1.2 μs (effective time, see Section S2.3 in the SI). In Figures 2 and S4, we plot the time series of the center–center distances for different base facet areas and show representative simulation snapshots. Videos of the simulation trajectories are available in the SI (Videos I, II, III, and IV).

**Figure 2:**
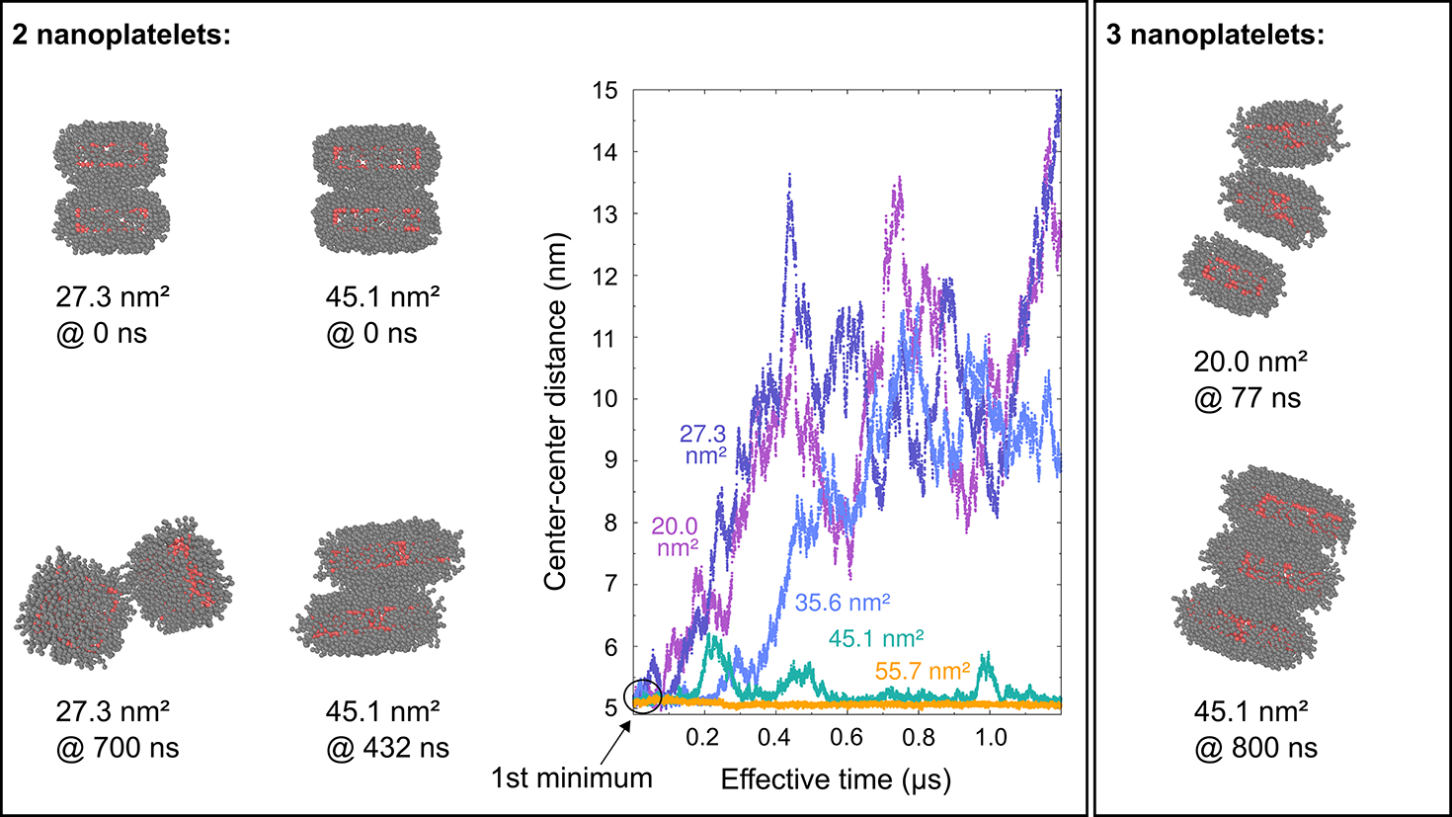
Snapshots of two and three nanoplatelet stacks with different facet areas from unconstrained molecular simulations, along with a time series of the center–center distances for stacks with two nanoplatelets. All nanoplatelets have the same thickness.

Nanoplatelets with a small facet area (≤ 35.6 nm^2^) rapidly move away from each other (Video I in SI). However, stacks of nanoplatelets with larger facet areas are stable. For facet areas equal to 45.1 nm^2^ and larger, no separation is observed (Videos II, III, and IV in the SI). While the platelets are stably bound to each other, they exhibit lateral displacements relative to the main axis of the stack and some rotate. These movements of the platelets are the cause of the fluctuations in the center-to-center distance of the platelets with 45.1 nm^2^ (see associated snapshot in Figure 2 and Video II). The behavior of the nanoplatelets is similar to that of micrometer-sized discs, which form stacks under the influence of depletion forces.^44^

In the next step, we perform simulations of unbound nanoplatelets. We place the platelets 10 nm apart in the start configuration. Then, we allow the platelets to move freely for at least 8 μs (see Figures S3, S5, S6, and S7 in the SI). No agglomeration is observed regardless of the nanoplatelet base facet area. We attribute this to the limitation of the simulation time and the agglomeration time scale. In other words, the agglomeration process is a rare event and occurs on longer time scales than we can simulate here.^20^

To garner insight into the nanoplatelet interaction, we compute the free energy of the system with respect to the distance between the platelets. Due to the anisotropic shape of the nanoplatelets, the pair interaction depends on the relative orientation of the platelets. We assume that layer formation of the solvent, and thus the strength of the solvation forces, are maximal for a parallel base facet to base facet orientation of the platelets. Therefore, we mainly limit our analysis of the interaction to this orientation (see Figure 1 for setup).

To obtain the free energy curves, we use multiple window umbrella sampling constrained simulations^45^ that are postprocessed using the weighted histogram analysis method (WHAM).^45−48^ In these constrained simulations, we do not allow the platelets to rotate or to move in *x* or *y* directions (see Section S2.4 in the SI for further details).

Unless otherwise specified, we study platelets with a 35.6 nm^2^ base facet area. This facet area is large enough to show the typical platelet behavior, see below, while at the same time, it is small enough that we can limit the simulation box size and the number of solvent molecules in the simulation (Table S7 in the SI).

We start by considering nanoplatelets without ligands. We find a strong oscillation of the free energy (Figure 3a) that is caused by solvation forces. The first minimum of the free energy is at 2.14 nm, a distance at which no solvent molecule fits between the two platelets. However, the transition between the first and the second minimum cannot be well sampled due to jamming.^1^ Therefore, the plot starts with the second minimum. Free energy minima are found where an integer number of solvent bead layers can fit within the two surfaces, separated by maxima for half-integer number of solvent layers. This can be visualized by directly computing the number of solvent molecules between the platelets (Figure 3c), where this layering manifests in step-like behavior for small distances. Figure 3d shows the solvent bead density of the system near the third minimum, where two layers of solvent beads between the platelets can readily be identified. Qualitatively, these results agree well with that of previous experiments and simulations.^7,49,50^

**Figure 3:**
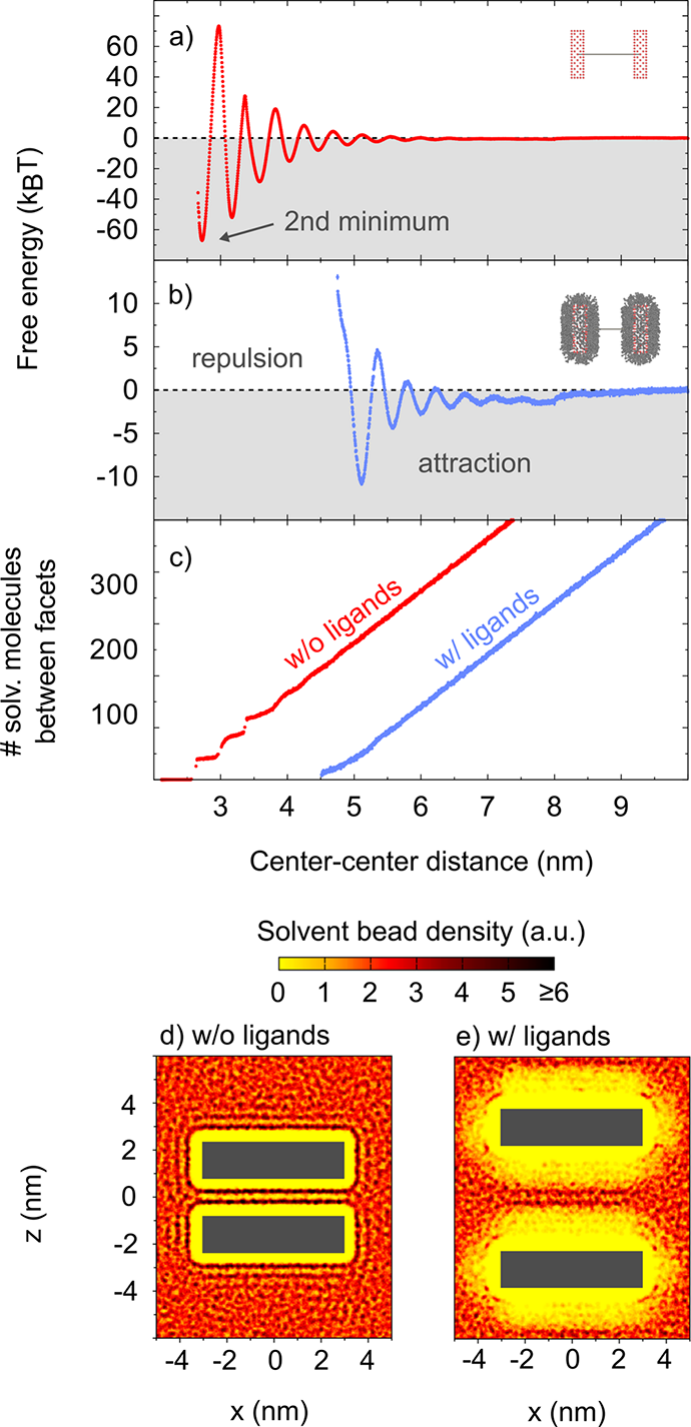
Results for square platelets with 1.5 nm thickness and an 35.6 nm^2^ base area: (a,b) Free energy curves obtained without ligand shell (panel a, red curve) and with ligand shell (panel b, blue curve). Error bars are smaller than the data points. (c) Number of solvent molecules between the nanoplatelet facets without ligands (red curve) and with ligands (blue curve). (d,e) Averaged solvent densities obtained without ligand shell (panel d) and with ligand shell (panel e). The dark gray boxes mark the positions of the platelets. Their average center–center distance is 3.17 nm in panel d and 6.03 nm in panel e.

In the next step, we examine the effect of the ligand shell on the interaction (Figure 3b).^17−19,51−56^ The distances at which extrema occur shift, as the platelets have an effectively larger thickness due to the ligand shell. However, the spacing between extrema remains constant. In agreement with experimental force measurements of alkane solvents between mica surfaces,^49^ the spacing between two minima or between two maxima is about the thickness of the alkane chain, in our case the size of a MARTINI bead.

The addition of the ligand shell results in a “softer” surface, which reduces the interaction strength (Figure 3b). Solvent molecules can permeate the end of the ligand brush, which results in absence of step-like behavior when the number of solvent molecules between the platelets is quantified (Figure 3c). Reduction of the solvent layering effect is also directly observed in the solvent bead density (Figure 3e), where we observe more diffuse solvent layers as compared to the distinct layers observed in Figure 3d. While the solvent molecules between two platelets in absence of a ligand shell show a strong tendency to orient parallel to the surface, the solvent molecules between two platelets with a ligand shell show a nearly random orientation (see Section S1.5 in the SI), indicating a lower entropic penalty.

The “soft” surface leads to a much weaker layer formation. Since solvent molecules can move more freely and with a lower entropic penalty, the height of the free energy maxima in the transition between two minima is reduced, and the free energy minima become shallower, both relative to the free energy at infinite separation. In other words, the free energy barriers between minima are reduced in height. This is a general trend we observe in our simulations: the “softer” the surface, the weaker solvation forces are.

Dense ligand shells consisting of a brush of shorter ligands correspond to “hard” surfaces, while longer ligands correspond to “soft” surfaces. The choice of the ligand length therefore enables control over solvation force strength. Additionally changing the ligand length allows to tune the distance between platelets in stacks (see Section S1.6 in the SI).

We also observe this behavior of “hard” versus “soft” surface for the ligand grafting density. For a higher grafting density, we obtain a “harder” surface, and the interaction is stronger. For a lower grafting density, we obtain a “softer” surface, and the interaction becomes weaker (see Section S1.7 in the SI for further discussion).

Changing the thickness of the platelets has little effect on the interaction. The interaction between thicker platelets becomes slightly more attractive (see Section S1.8 in the SI).

As already observed above, a crucial factor is the base facet area of the interacting platelets. We calculate free energy curves of ligand coated platelets with various base facet areas, ranging between 2.2 and 55.7 nm^2^ (see Table S11 and Figure S18 in the SI). For the two smallest sizes (2.2 and 5.0 nm^2^), the layering effect and the solvation forces vanish. The free energy curves of all larger platelets have a similar shape as the calculation in Figure 3b. Since the thickness of the ligand shell does not change with the size of the facets, the distances at which the maxima and minima of the free energy are found do not change either. However, the larger the area of the interacting facets is, the stronger the interaction.

To quantify this effect, we plot the values of the free energy at the minima and maxima as well as at a distance of 4.75 nm that corresponds to significant brush repulsion (Figure 4). Both the attraction at the minima and the repulsion at the barriers’ maxima (including the distance of 4.75 nm) increase with facet area. How strongly the free energy changes with the facet area depends on the distance between the nanoplatelets. For the lower distance minima and maxima, the slope is steeper than for the higher distance minima and maxima. We find a linear behavior for the free energy of attraction and repulsion for larger facet areas. The linear trend starts at around 20.0 nm^2^ facet area for the attraction at the minima, while it starts at around 27.3 nm^2^ facet area for the repulsion at the maxima.

**Figure 4:**
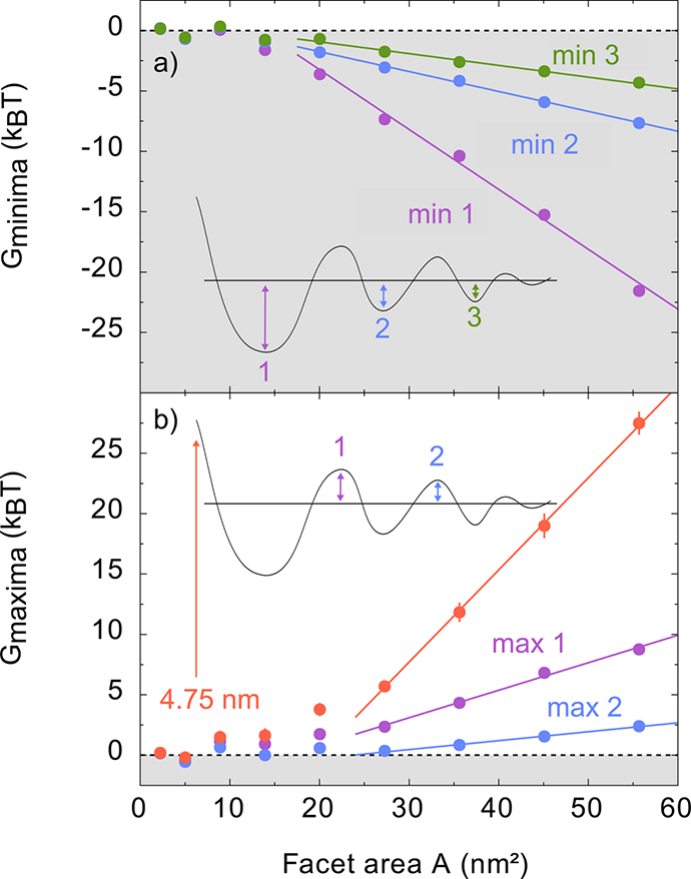
Variation of the facet size leads to a change in the strength of the interaction. Plotted are the depth of the free energy minima (panel a) and the height of the free energy maxima together with the free energy at 4.75 nm distance (panel b) relative to the free energy at infinite separation. In panel a, linear fits are attached to points equal to and larger than 20.0 nm^2^ and in panel b to points equal to and larger 27.3 nm^2^.

The linear trends for the minima are in line with expectations. There are mainly two effects causing the attraction within our system: the van der Waals attraction between the ligand shells and an excluded volume/depletion effect of the solvent. The van der Waals attraction should scale with the number of ligand molecules and therefore with the facet area. The depletion effect due to the solvent scales with the excluded volume, similar to the depletion effect between larger particles caused for example by polymer coils.^1,57,58^ The minima are always at the same position, since it is determined only by the thickness of the ligand shell, which does not change with the facet area. Hence, the volume is proportional to the area. In conclusion, the free energy of a minimum scales proportional to the facet area:
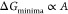
1Similar arguments can be used to motivate the linear trend of the repulsion. The compression of the ligands and the effect of the mixing of the solvent with the ligands are likely to make the most significant contribution at 4.75 nm distance between the nanoplatelets.^2,3^ Another effect is the increase in excluded volume in the transition between two minima. These effects will scale with the facet area:
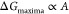
2

For smaller nanoplatelets the linear behavior is not observed, which we attribute to the change in ligand density and to the deformation of the ligand shell. As the nanoplatelet size is reduced, the length of the ligands becomes comparable to the nanoplatelet dimensions, and the overall shape of the nanoparticle becomes similar to a sphere (see Figures S19 and S20 in the SI). The ligand shell “softens”, and the solvation forces weaken faster.

We can conclude that solvation forces are responsible for the stabilization of nanoplatelet stacks that we observe in the unconstrained simulations at larger facet areas (Figure 2). The comparison of the free energy curve with the unconstrained simulations shows that the first minimum in the free energy curve in Figure 3 is the global minimum and the thermodynamically favored state. However, to fully understand the nanoplatelet stacking behavior, we have to take into account the dependence of the pair interaction on the relative orientation of the nanoplatelets. The barriers we find in the free energy curve are an upper bound, as assembling in the direct parallel base facet to base facet orientation is probably the most unfavorable assembly pathway. The free energy barriers are likely to be lower for more realistic assembly pathways where the nanoplatelets can rotate freely. For example, if two platelets approach in a parallel orientation and slide along each other in the global minimum, the barriers vanish (see Figure S22 in the SI).

Regarding the unconstrained simulations, the barriers due to the solvation forces explain why the platelets do not self-assemble immediately in the unconstrained simulations. We assume that two platelets meeting in the right orientation where they can self-assemble is a rare event. This is probably the reason why we do not observe self-assembly on the time scale of our unconstrained simulations (ns to μs). However, this is a different matter on the time scales of a typical experiment that are much longer. For example, the precipitation dynamics observed by Jana et al. takes place on time scales of minutes or hours.^20^

The ability to overcome these orientation dependent barriers is one of the reasons why CdSe nanoplatelets form stacks, as opposed to other types of nanoplatelets which are kinetically trapped (see Section S1.10 in the SI).^59−61^

In summary, we conclude that solvation forces, dependent on properties like ligand length, ligand grafting density, and facet area, can replace the van der Waals attraction between colloid nanoparticles as dominant attractive force in some systems. In a good solvent, the ligand shell serves not only to reduce the strength of the van der Waals attraction between the nanoparticle cores by increasing the distance between the nanoparticles but also to tune the solvation forces.

In the case of nanoplatelets, solvation forces can significantly contribute to the stack formation and the precipitation kinetics in apolar solvents. While CdSe nanoplatelets are a special case in terms of their weak van der Waals attraction and their large facet area relative to their particle size, our results demonstrate the importance of solvation forces for the interaction and steric stabilization of faceted nanoparticles in general.

Other effects like the addition of a nonsolvent,^62^ the addition of depletants,^20,63^ or the evaporation of the solvent^24^ can also influence the precipitation dynamics. Further studies are needed to clarify the interplay between these effects and the solvation forces.

## Data Availability

Partial data supporting the results reported in this paper are openly available at Zenodo^64^ (DOI: 10.5281/zenodo.7385599). Further data and simulations trajectories are available upon request from the authors.

## Supporting Information Available

Further computational details and results, including, for example, the solvent density, the solvent orientation, ligand grafting density/lattice constant, and the variation of the ligand length (PDF)
Video I shows the separation of two small platelets with 20.0 nm^2^ facet area (AVI)
Video II shows the movements of two attached, nonseparating nanoplatelets with 45.1 nm^2^ facet area (AVI)
Video III shows the movements of two attached, nonseparating nanoplatelets with 55.7 nm^2^ facet area (AVI)
Video IV shows the movement of a platelet stack consistent of three platelets with 45.1 nm^2^ facet area (AVI)

## Supplementary Material

nl2c02848_si_001.pdf

nl2c02848_si_002.avi

nl2c02848_si_003.avi

nl2c02848_si_004.avi

nl2c02848_si_005.avi
