# Supplementary material for: The Crucial Role of Solvation Forces in the Steric Stabilization of Nanoplatelets
Source: Nano Lett. 2022 Dec 9;22(24):9847–53. doi: 10.1021/acs.nanolett.2c02848 (PMC9801426; doi:10.1021/acs.nanolett.2c02848)
Supplement: Supplementary file 1 — nl2c02848_si_001.pdf [file nl2c02848_si_001.pdf]

Supporting Information for: The Crucial Role of Solvation Forces in the Steric Stabilization of Nanoplatelets

Nanning Petersen,† Martin Girard,† Andreas Riedinger,*,† and Omar Valsson*,†,‡

†*Max Planck Institute for Polymer Research, Mainz, D-55128, Germany*

‡*Department of Chemistry, University of North Texas, Denton, TX 76201, USA*

E-mail: riedinger@mpip-mainz.mpg.de; omar.valsson@unt.edu

Contents

S1 Additional results **2**

S1.1 Estimation of the van der Waals interaction between the CdSe nanoplatelets 2

S1.2 Comparison of the MARTINI and the united atom TraPPE-UA force fields 5

S1.3 Nanoplatelet stack stability 8

S1.4 Solvent bead density 14

S1.5 Solvent orientation 15

S1.6 Effect of ligand molecule length 17

S1.7 Ligand grafting density and lattice constant 21

S1.8 Nanoplatelet thickness 25

S1.9 Nanoplatelet facet area 26

S1.10 Orientation Dependence of Nanoplatelet Interactions 30

S2 Methods **35**

S2.1 Model System Setup 35

S2.2 Choice of ligand grafting density for the simulations 36

S2.3 Molecular Dynamics Simulations 38

S2.4 Free Energy Calculations 38

S2.5 WHAM parameter 40

S2.6 Base facet area, solvent number and average box size 44

S2.7 Simulation setup for infinitly large platelets 47

S2.8 Data availability 48

References **48**

S1 Additional results

S1.1 Estimation of the van der Waals interaction between the CdSe nanoplatelets

We use the Hamaker approximation to estimate the van der Waals interaction energy between the crystals of CdSe nanoplatelets in alkane solvents.

A problem of this approach is, that at contact of the two ligand shells, the van der Waals interaction should be determined mainly by the ligand shell and not by the solvent. The Hamaker constant of the ligand shell is unknown. However, we assume, due to the chemical similarities between the ligand tails and the alkane solvent, that an alkane solvent is a good approximation of the ligand shell.

The Hamaker constant of CdSe, according to Rabani, is $\mathrm{A}_{\mathrm{CdSe}} = 6.216\ 10^{-20}$ J.[1] Hough and White have calculated Hamaker constants A_{Alkane} for alkanes with different chain length (see Table S1).[2] We calculate the Hamaker constant of CdSe interacting through an alkane

solvent by:

$$A_{\text{CdSe-Alkane-CdSe}} \simeq \left(\sqrt{A_{\text{CdSe}}} - \sqrt{A_{\text{Alkane}}}\right)^2. \tag{S1}$$

With this result, we approximate the interaction energy between two nanoplatelet facets by:

$$G_{\text{van der Waals}} = -\frac{A_{\text{CdSe-Alkane-CdSe}}}{12\pi d^2} S_{\text{surface}}, \tag{S2}$$

where d is the distance between the surfaces of the two nanopatelets, and S_{surface} is the interacting surface area.[3] We should note, that Equation S2 describes the interaction between two infinitely thick flat surfaces. It can therefore be assumed that the equation overestimates the strength of the van der Waals attraction.

In Figure S1a we compare the effect of different alkane lengths on the face to face interaction energy for two nanoplatelets with 225 $\mathbf{nm^2}$ facet areas. The attraction between the nanoplatelets reduces with the chain length of the alkane solvent molecules. In Figure S1b we compare the van der Waals attraction for different facet areas. The larger the facet area is, the stronger is the attraction between the nanoplatelets.

Jana *et al.* have measured the face to face distances between stacked nanoplatelets with in average 225 nm^2 facet areas for different ligands. They have found surface to surface distances between 2.1 and 4.1 nm.[4] According to our estimations, the attractive van der Waals interaction energy for this platelet size lies around or below 1 $k_{\text{B}}T$

Although we do not know exactly what effect the ligand shell has on the van der Waals interaction and which alkane solvent describes the ligand shell better, we conclude from these estimations that the van der Waals interaction between the two CdSe crystals of the nanoplatelets is too weak to be (alone) responsible for the tendency of nanoplatelets to form stacks in solution.

This may be surprising, as the stability of colloid particles/nanoparticles in apolar solutions is often estimated via the attractive van der Waals interaction, and the repulsiv contribution of the ligands.[5] However, metals have significantly larger Hamaker constants

(20 to 50 10^{-20} J).[3,6] Therefore, while for gold or silver the van der Waals forces dominate the overall interaction between the nanoparticles or at least make a large contribution, they are comparable small for CdSe (see Figure S1c).

Table S1: Hamaker constants of alkane solvents with different molecule chain lengths at 20°C from Ref.[2]

Alkane chain length	Hamaker constant (10^{-20} J)
5	3.75
6	4.07
7	4.32
8	4.50
9	4.66
10	4.82
11	4.88
12	5.04
13	5.05
14	5.10
15	5.16
16	5.23

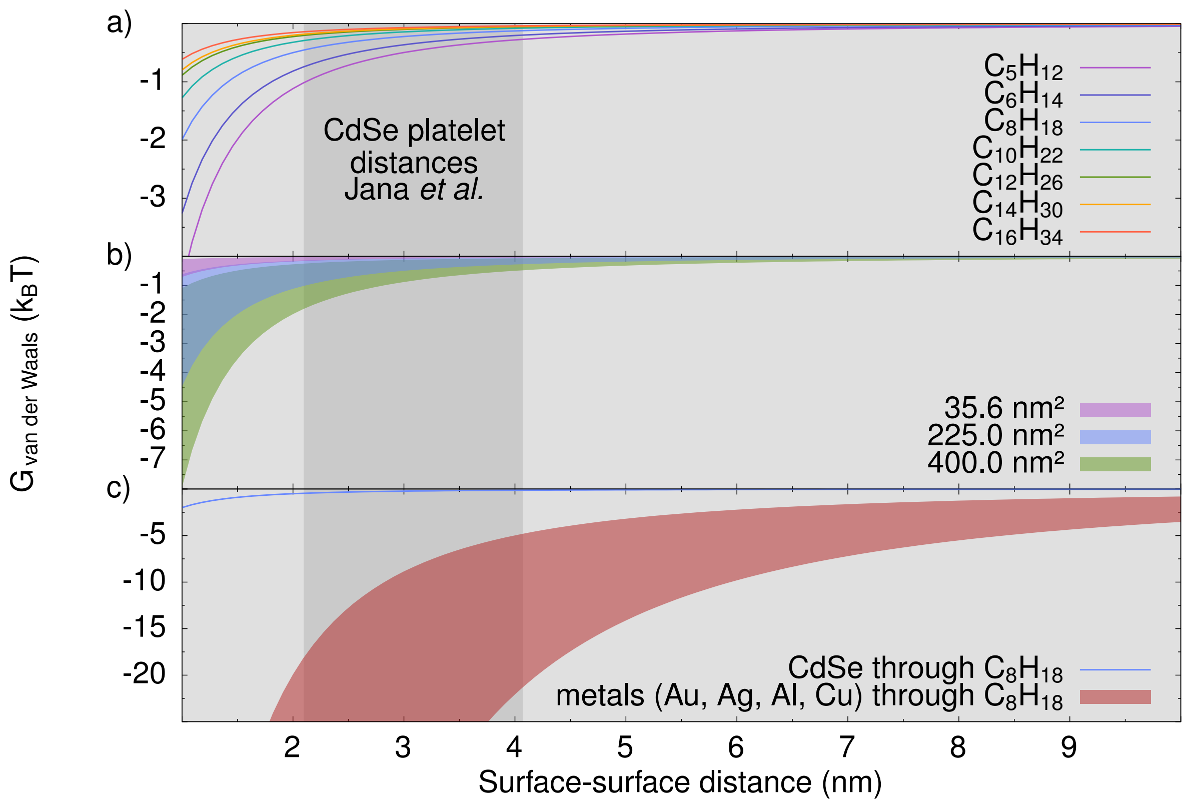

Figure S1: Estimation of the van der Waals interaction between CdSe nanoplatelets. In a) we compare the interaction through different alkane solvents for a facet area of 225 nm^2, and in b) how the energy changes for different facet areas. In b) we illustrate the effect of the different solvents (propane to hexadecane) by broader curves. The overlapping of curves leads to a change in their colours. In c) we compare the van der Waals interaction of CdSe nanoplatelets with hypthetic nanoplatelets consisting of metal. The solvent is octane, and the facet area 225 nm^2. The gray rectangle between 2.1 and 4.1 nm marks CdSe nanoplatelet distances for a facet area of 225 nm^2, and different ligands measured by Jana *et al.*[4]

S1.2 Comparison of the MARTINI and the united atom TraPPE-UA force fields

For the description of the ligands and the solvent molecules in the analysis of the pair interaction between the CdSe nanoplatelets, we mainly use the MARTINI model.[7] In this model, each bead represents four heavy atoms, such as C, N, or O. The description with the Martini model reduces the number of beads in the simulations. The resulting reduction in the required computational capacity allows us to consider the pair interaction in more detail.

We can analyze their dependence on different parameters, such as platelet size, ligand length, and ligand grafting density. The disadvantage of this approach is that chemical details are lost as the resolution is reduced.

In this section, we compare the MARTINI force field with the TraPPE-UA force field.[8] The TraPPE-UA force field is a united atom force field, i.e. heavy atoms, such as C, are represented as a bead together with the hydrogen atoms to which they are connected. Thus, the TraPPE-UA force field comes closer to an all atomistic description.

Since the number of beads in a united atom description of ligands and solvent molecules increases greatly, we decided to use a simplified approach for this comparison. Instead of analysing the interaction between two platelets, we study the interaction between the ligand shell of an infinite large platelet / surface with the solvent. Figure S2b shows a snapshot of the setup. The solvent is octane, as in all following MARTINI simulations. The ligands consist of 16 CH_x beads, corresponding to the 4 C1 MARTINI beads we mostly use. The ligand grafting points are arranged in a face centered lattice with a lattice constant of 0.608 nm, as found on the surface of CdSe nanoplatelets.[9] We give a detailed description of the setup, and the TraPPE-UA force field we use in Section S2.7.

We create a similar setup for the MARTINI force field (Figure S2c). Here, the ligands consist of four C1 MARTINI beads. The ligand grafting points are also arranged in a face centered lattice. However, we use the lattice constant 0.746 nm.

This means that in the TraPPE-UA setup the ligand density is 5.4 ligands / nm^2, while in the MARTINI setup it is only 3.6 ligands / nm^2. Experimental measured densities are close to the value we use in the TraPPE-UA setup.[10,11] However, as the C1 MARTINI beads are quite large, we have to choose a larger lattice constant / lower ligand grafting density for this setup (see Section S2.2 for further discussion).

Crucial to the strength and expression of the solvation forces, and thus the interaction between the nanoplatelets, which we consider in this study, is the layering of the solvent molecules at the interface of the ligand shell. To investigate the layering of the solvent, we

calculated the solvent and ligand densities in each of the two systems (Figure S2a). The densities in both systems show similar features. In both cases, ligand and solvent densities overlap, and the solvent density oscillates near the solvent-ligand interface.

We can conclude from this comparison that layer formation is well described in both setups. We therefore conclude that our setup with the MARTINI model is well suited to study the pair interaction among the CdSe nanoplatelets. We expect that the layer formation, and interaction between the platelets will be well described qualitatively, while there will be quantitative differences in the strength of the interaction.

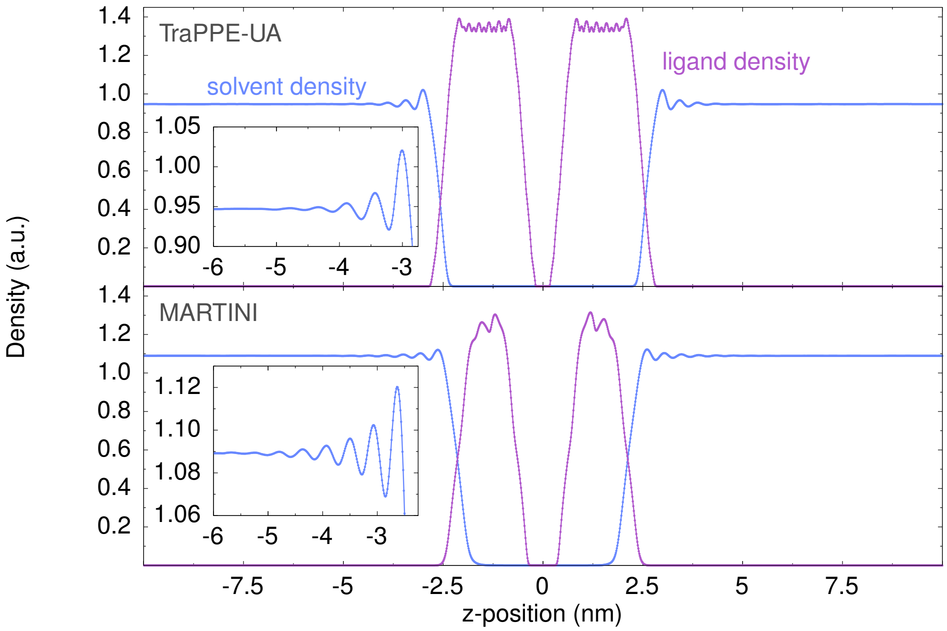

(a)

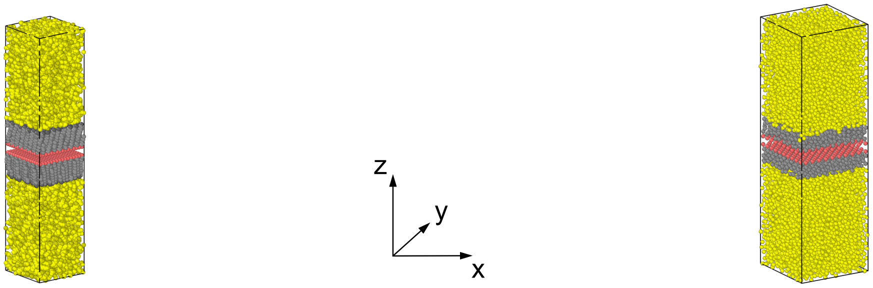

(b) TraPPE force field

(c) MARTINI force field

Figure S2: Comparison of the ligand and solvent densities as a result from the setup with the TraPPE force field, and the MARTINI force field (panel **a**). A snaphshot of the simulation box from the TraPPE setup is shown in panel **b**, and a snapshot from the MARTINI setup in panel **c**. In both snapshots are the solvent beads in yellow, the ligand beads in gray, and the surface beads in red. In the snapshot of the TraPPE setup, the CH_3 beads have a darker color than the CH_2 beads.

S1.3 Nanoplatelet stack stability

Supplementary trajectories of unconstrained simulations are plotted in Figures S3-S5.

For simulations with an initial state in which nanoplatelets are not assembled, we calcu-

late the mean square displacements:

$$\mathrm{MSD}(\tau) = \frac{1}{\#_{\mathrm{platelets}}} \sum_{j=1}^{\#_{\mathrm{platelets}}} \frac{1}{N} \sum_{i=1}^{N} \left(\vec{R}(t_i + \tau) - \vec{R}(t_i) \right)^2 . \tag{S3}$$

$\#_{\mathrm{platelets}}$ is the number of platelets, t_i are timesteps, τ the lag time and N is the number of reference frames taken into account. N varies dependent on the trajectory length and τ. The mean square displacements from simulations with two nanoplatelets can be found in Figure S6 and from simulations with three nanoplatelets in Figure S7.

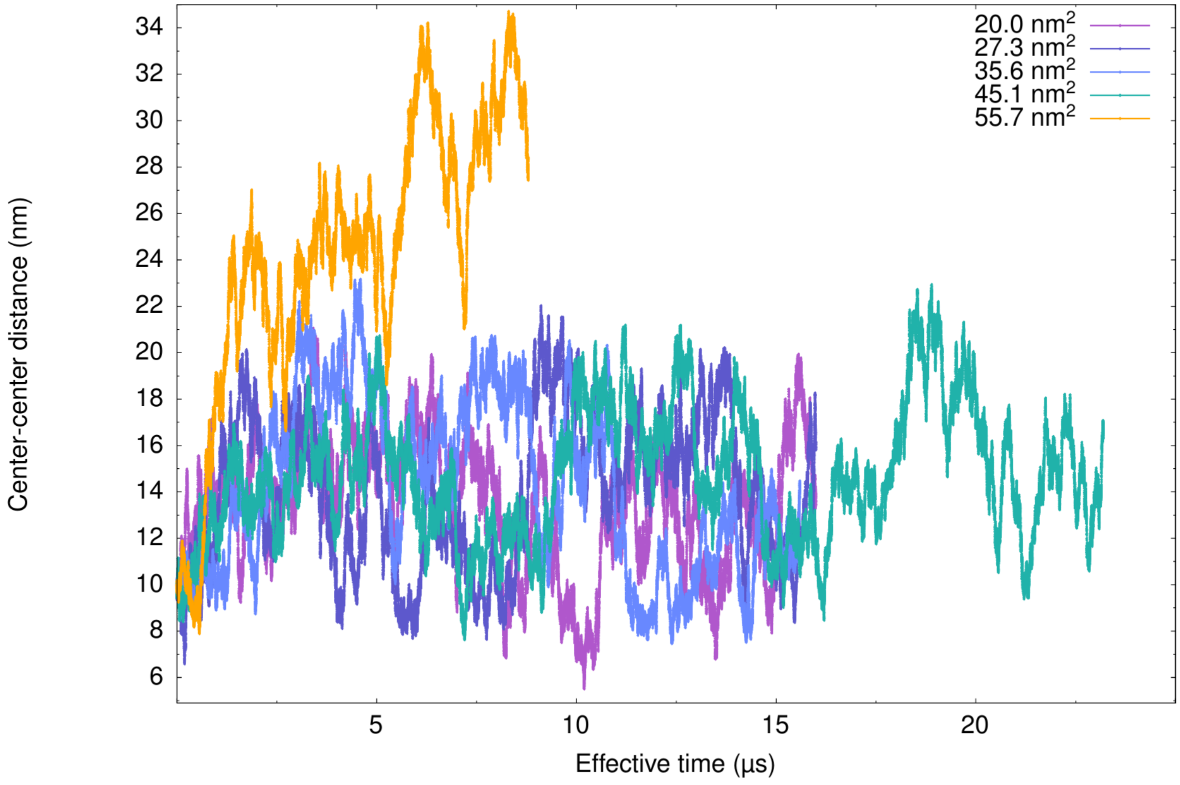

Figure S3: Time series of the center-center distances for two nanoplatelets. Simulations are initiated in an not assembled state. The facet surface is varied. 55.7 nm^2 behaves slightly different, since the simulation box is larger (see Table S5).

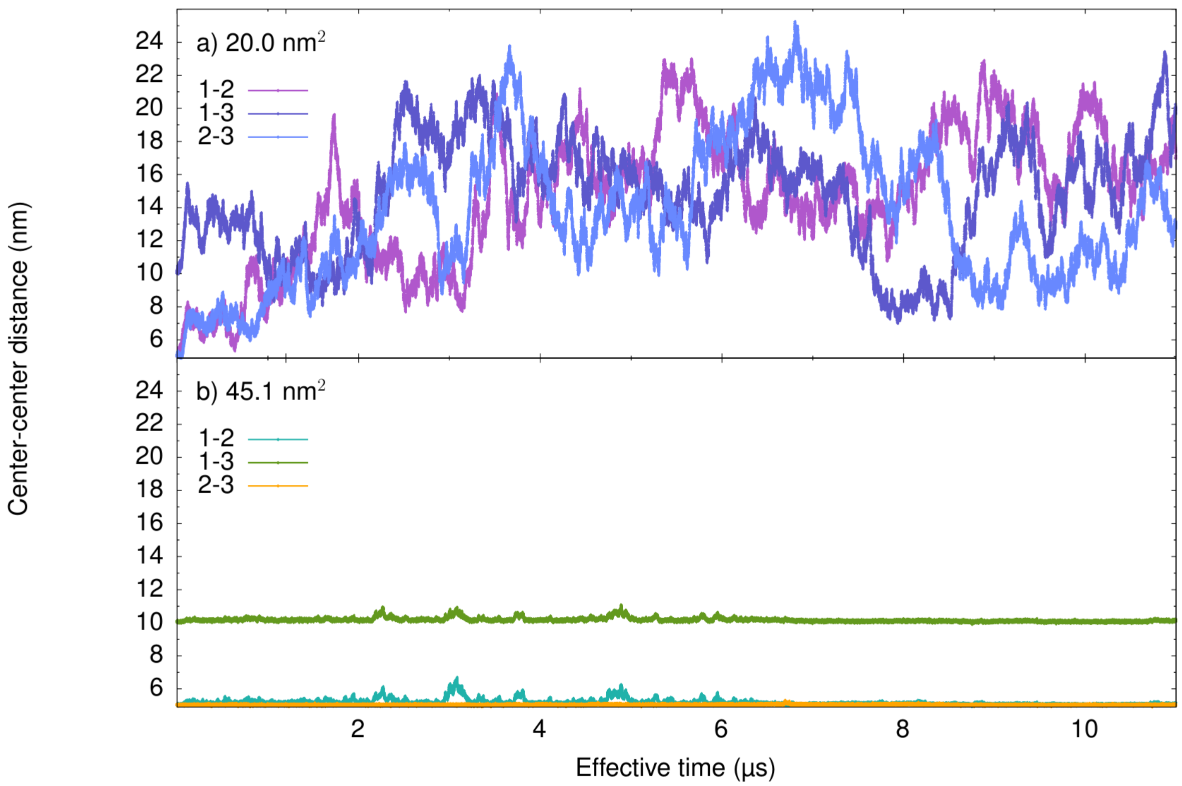

Figure S4: Time series of the center-center distances for stacks with three nanoplatelets (number 1,2 and 3 in the key of the panels). In panel a), the facet area is 20.0 nm^2 and in panel b) it is 45.1 nm^2

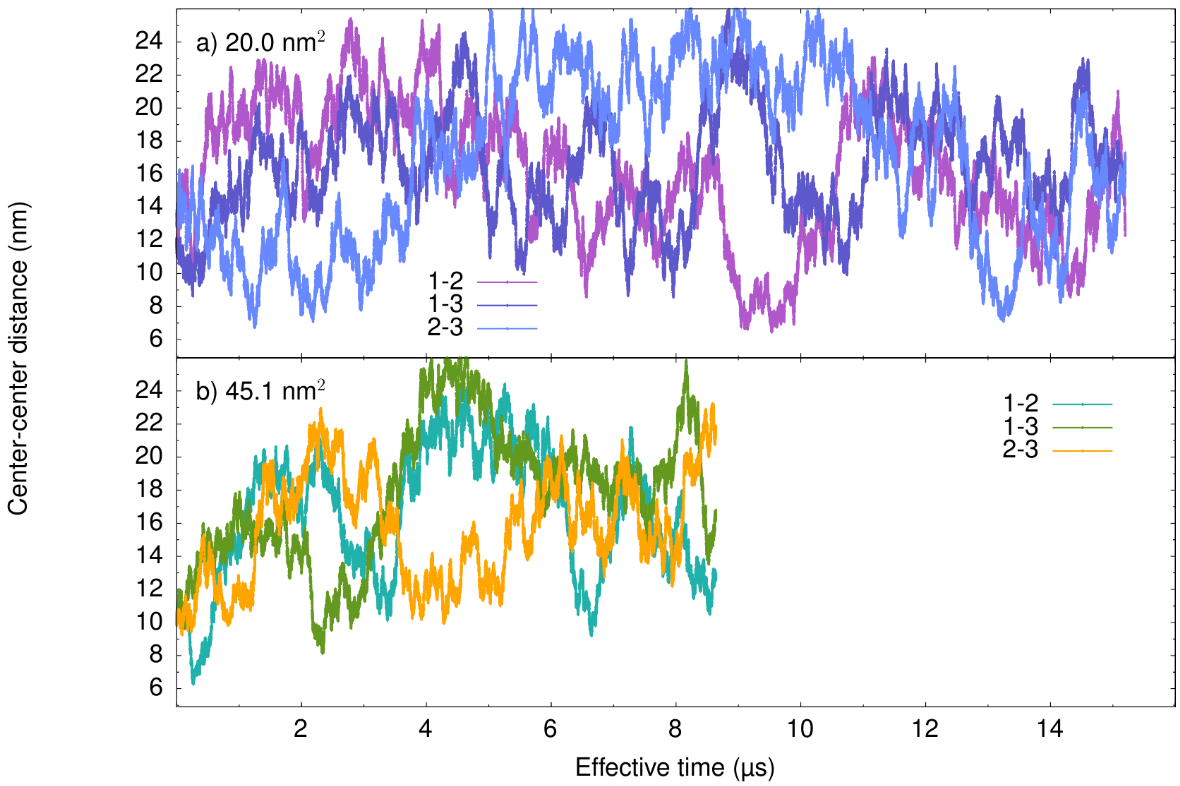

Figure S5: Time series of the center-center distances for three nanoplatelets (number 1,2 and 3 in the key of the panels). Simulations are initiated in an not assembled state. In panel a), the facet area is 20.0 nm^2 and in panel b) it is 45.1 nm^2

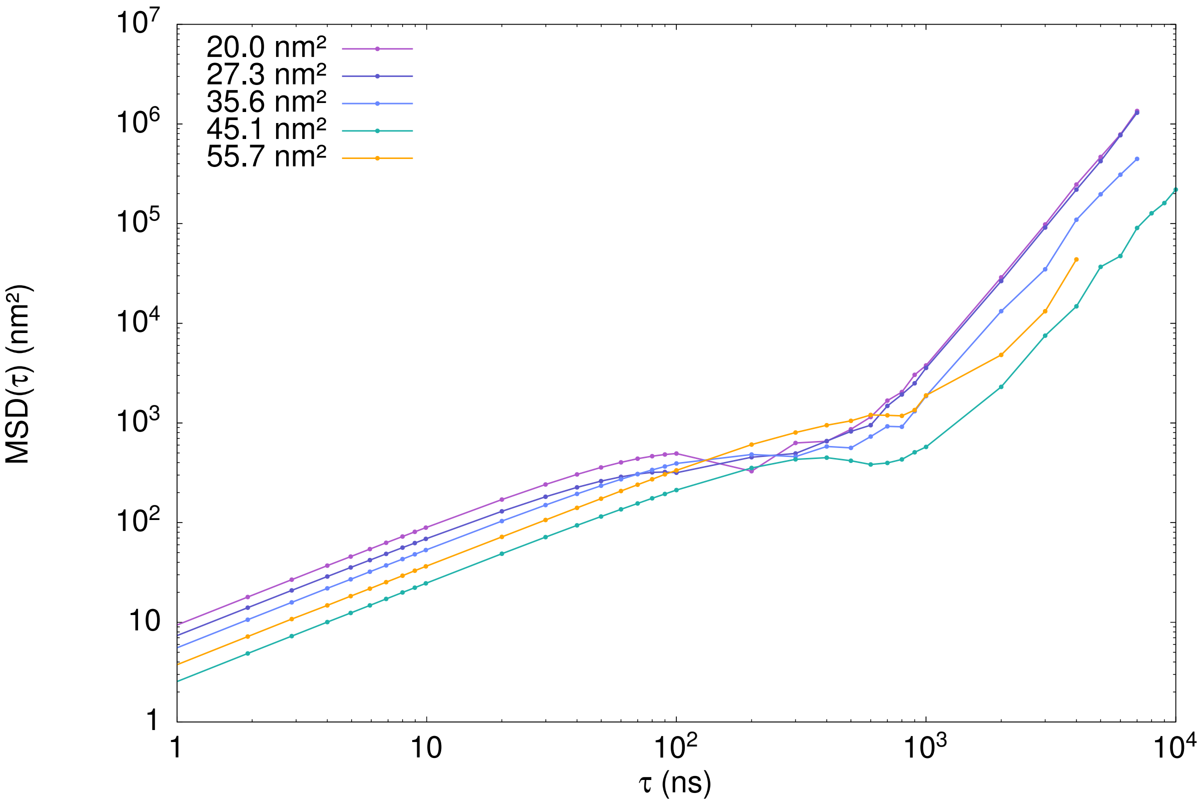

Figure S6: Mean square displacement of unconstrained simulations with two nanoplatelets, where nanoplatelets are initially not in contact. Self-assembly of nanoplatelets is not observed in these simulations.

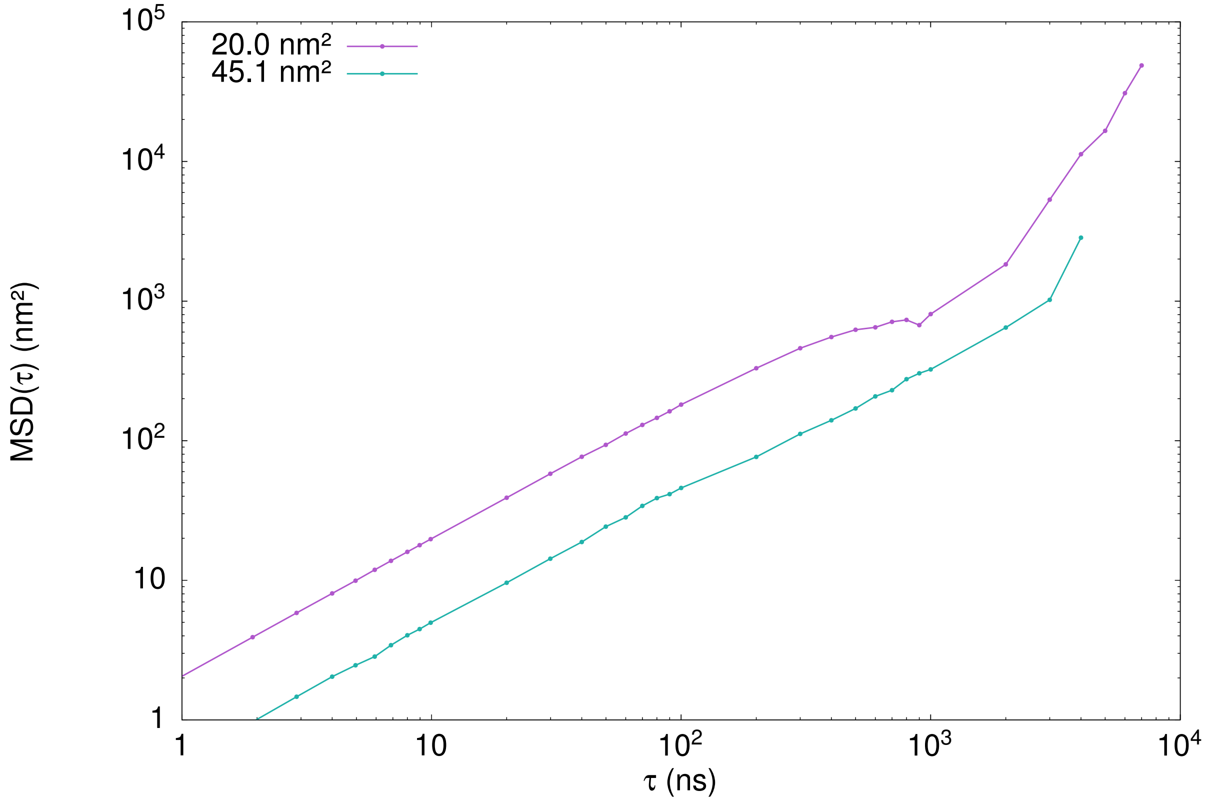

Figure S7: Mean square displacement of unconstrained simulations with three nanoplatelets, where nanoplatelets are initially not in contact. Self-assembly of nanoplatelets is not observed in these simulations.

S1.4 Solvent bead density

We create heatmaps of the solvent bead density at free energy minima (Figures 3 and S8). In a first step, a window close to a minimum is selected from the Umbrella calculation. We then remove the Umbrella potential, and relax the system. After the system is at the minimum, we record the trajectory over a period of time. From this trajectory we select equally distributed frames over which we determine the averaged solvent density (see Table S2). To create the solvent bead density heatmap of a frame, we bin space in the xz plane, using a bin-size of 0.025 nm. We then map beads that are within 0.076 nm of the plane to these bins. In order to smoothen the resulting histogram, we assign each solvent bead a radius of 0.1 nm. When building the histogram, particles count as being in all bins they touch. Finally, we average over the selected frames. This procedures create a time averaged histogram of where solvent beads are located.

Table S2: Summary of the number of frames and the time interval over which the density plots are averaged.

# of ligand beads	minimum	# of frames	time interval / ns
0	1	49	200
0	2	28	200
0	3	28	200
4	1	28	140
4	2	28	180
4	3	28	180

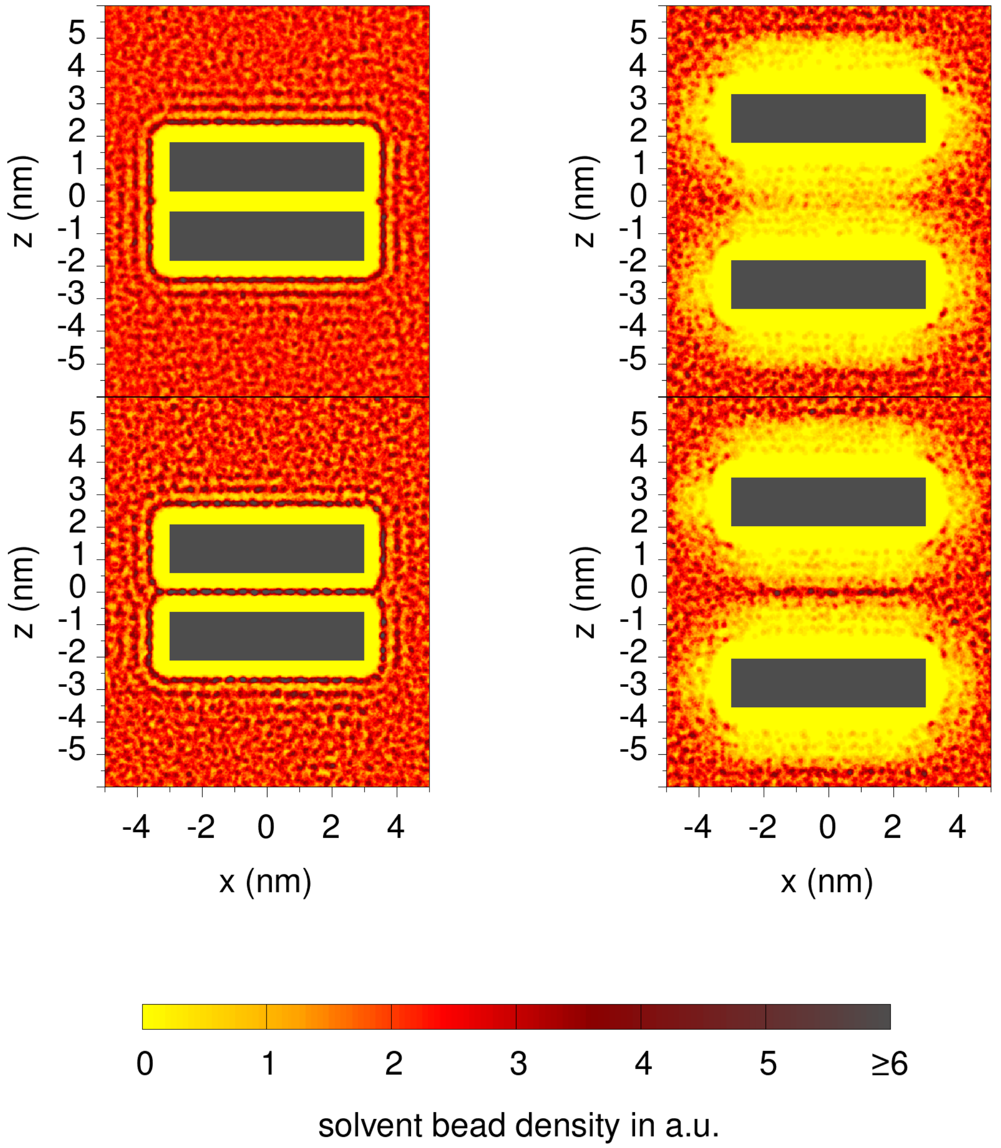

Figure S8: Heatmaps of the solvent bead density in the first (top) and second (bottom) minimum. We compare the solvent density for platelets without (left) and with (right) a ligand shell. The nanoplatelets have 35.6 nm^2 base facet area and 1.5 nm thickness. The average center-center distances in the first minima are 2.14 nm, and 5.11 nm. The distances in the second minima are 2.72 nm, and 5.58 nm.

S1.5 Solvent orientation

We calculate the nematic order parameter S to describe the orientation of the solvent molecules between the two surfaces of the platelets (Figure S9). In simulations without

a ligand shell, at positions of a free energy minimum, we find that the solvent molecules between the platelets show a tendency to orientate parallel to the platelet surface. This tendency vanishes with increasing platelet distance. For instance, at the third minimum we find that molecules are oriented perpendicular to the surface. Such molecules straddle the solvent layers, with one MARTINI bead in each of the first and second solvation layers (Figure S10). In contrast, in simulations with a ligand shell solvent molecules show a nearly random orientation distribution (Figure S9, S10).

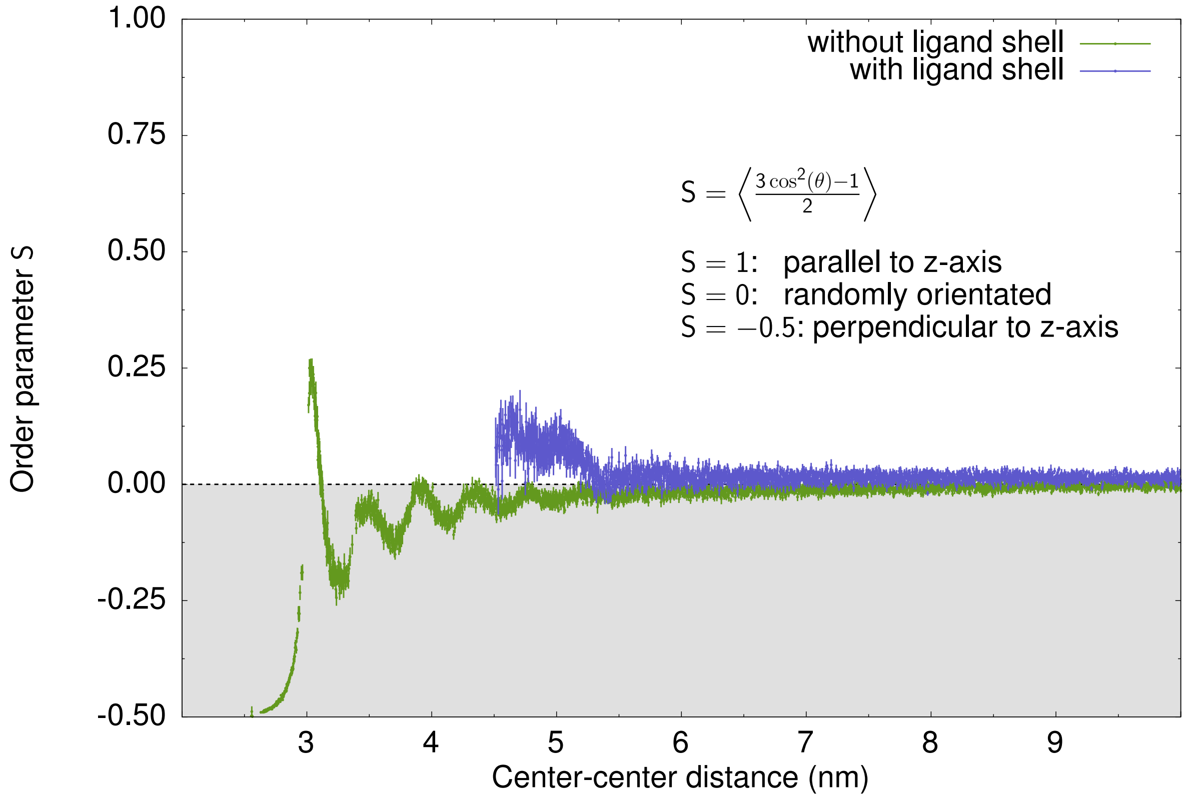

Figure S9: Nematic order parameter, describing the average orientation of the solvent molecules between the two platelets in a system without a ligand shell and in a system with a ligand shell. θ is the angle between the solvent bond and the axis perpendicular to the facet surface (z-axis). The platelets have 35.6 nm^2 base facet area and 1.5 nm thickness.

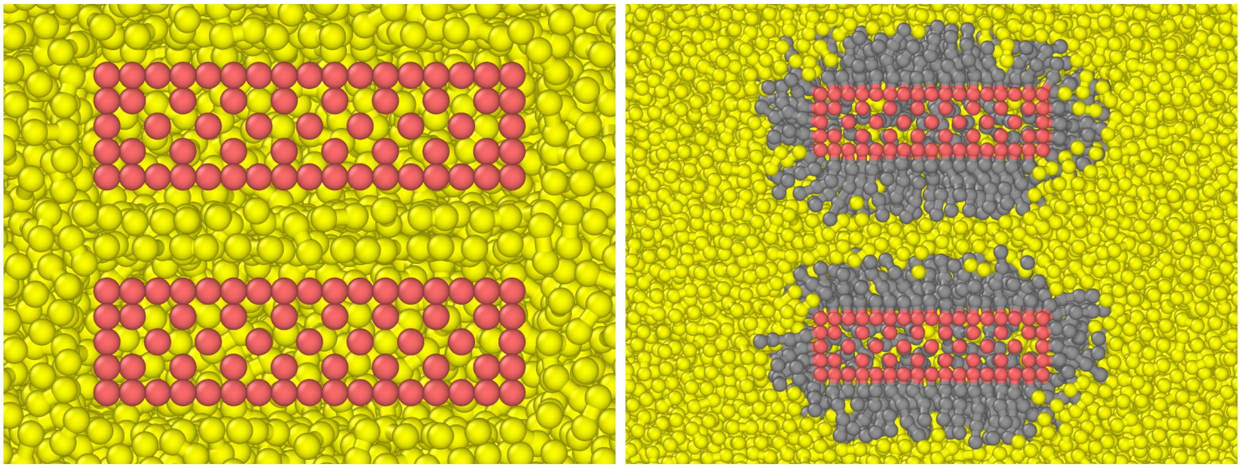

Figure S10: Snapshots of two nanoplatelets without (left) and with (right) a ligand shells in the third minimum. They illustrate the orientation of the solvent molecules. The platelets have 35.6 nm^2 base facet area and 1.5 nm thickness. CdSe beads are shown in red, ligand beads in grey, and solvent beads in yellow.

S1.6 Effect of ligand molecule length

The strength of the interaction between two platelet facets and the effective thickness of the platelets is influenced by the ligand length (Figure S11). Due to the change in effective thickness, the positions of the extremas are shifting with the ligand length. The shorter the ligands are, the thinner is the ligand shell and the closer is the distance, at which the global minimum lies. The strength of the interaction increases as the ligands become shorter (Figure S12). We relate this change in strength to the change in the "softness" of the ligand shell (Figure S13).

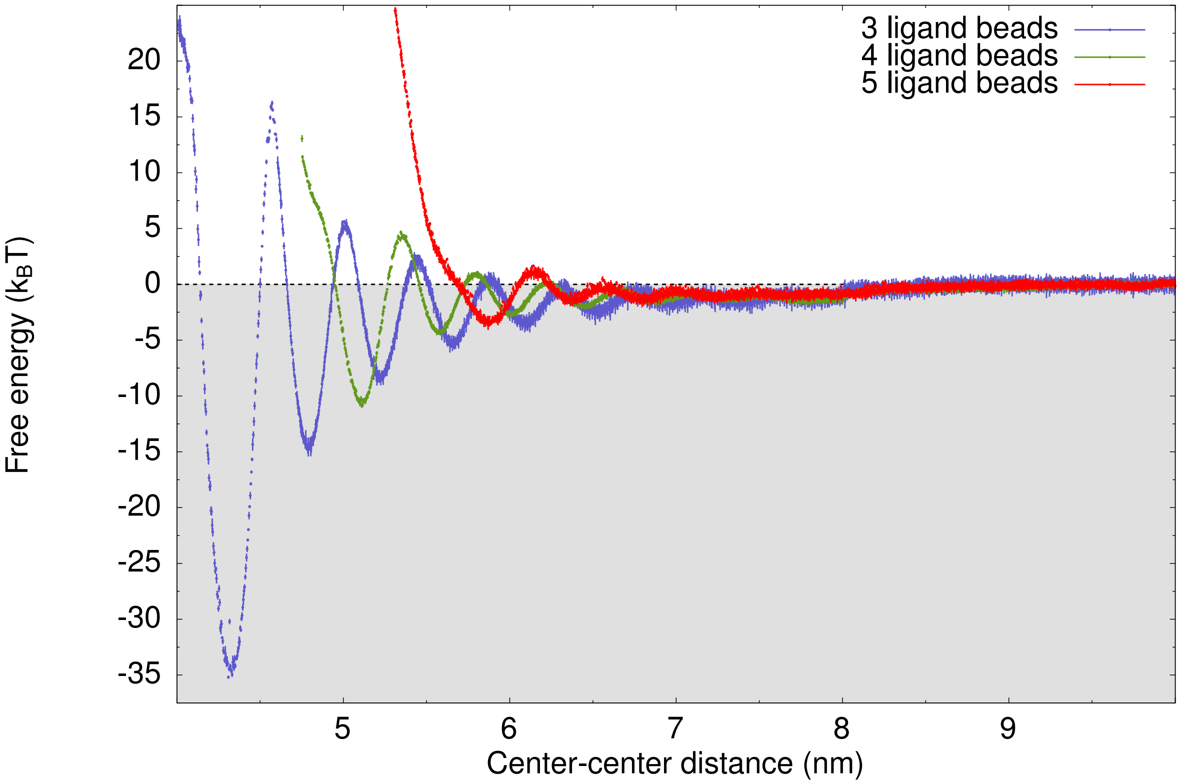

Figure S11: Effect of ligand molecules length. Free energy curves of base facet to base facet interaction are shown (error bars included). The platelets have 35.6 nm^2 base facet area and 1.5 nm thickness. The length of the ligand molecules is varied. The curve of the calculation without ligand shell is not shown, because it would exceed the scale (see main publication, Figure 3).

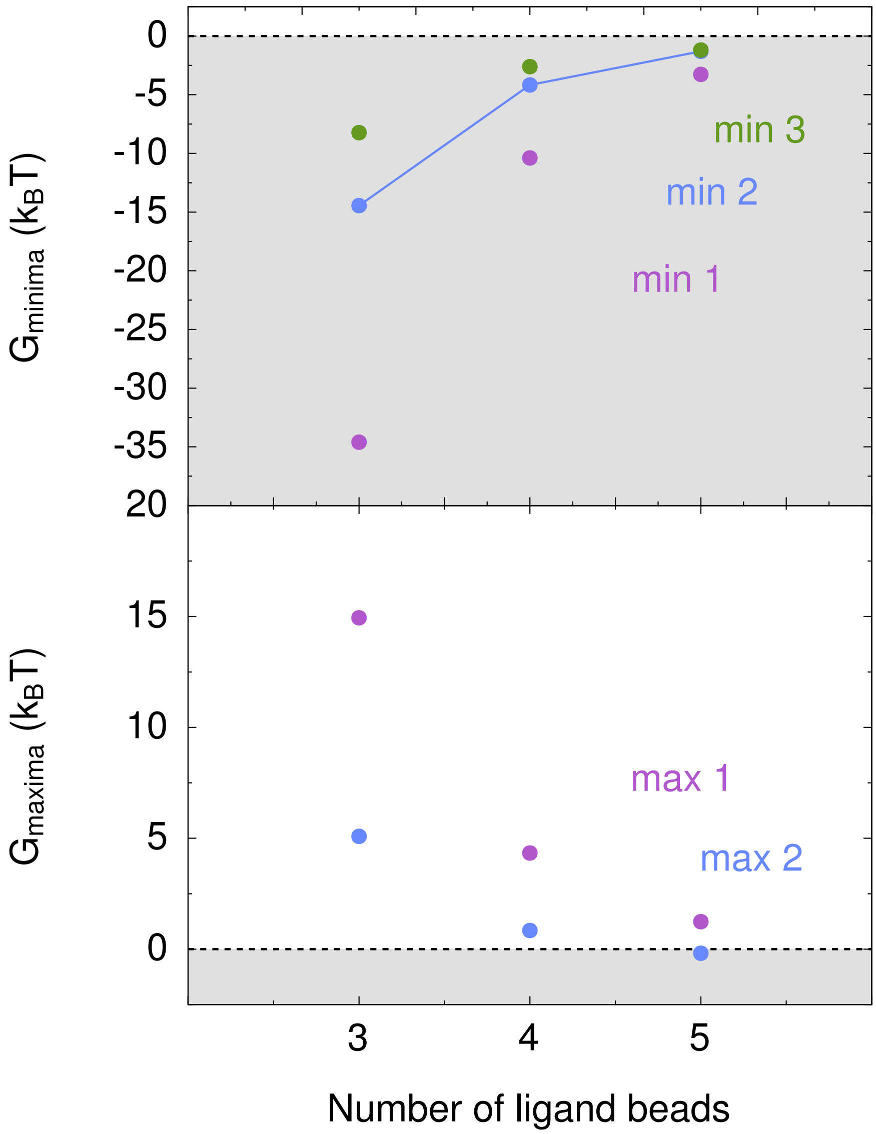

Figure S12: The variation of the ligand length leads to a change in the strength of the interaction. Plotted are the depth of the free energy minima (panel **a**), and the height of the free energy maxima (panel **b**). In panel **a** we connect the points of minima 2, as one point lies behind a point of minima 3. The error-bars of the fits are smaller than the points.

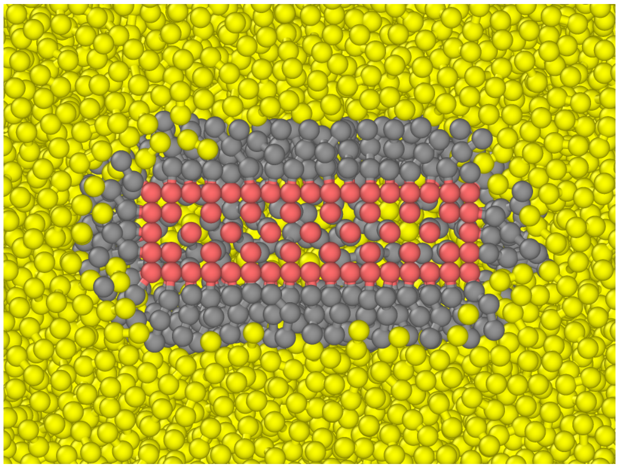

(a) 3 MARTINI beads

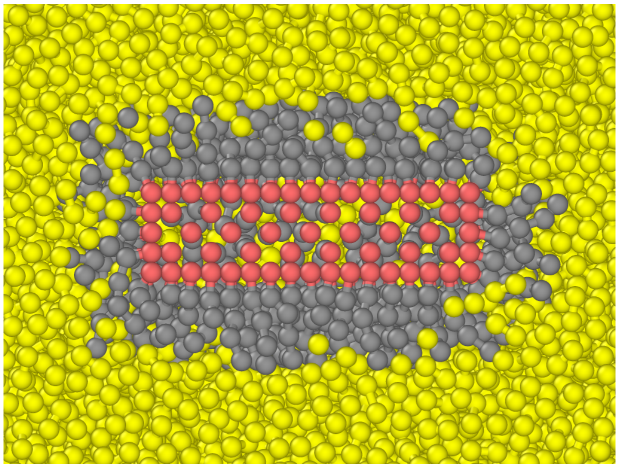

(b) 4 MARTINI beads

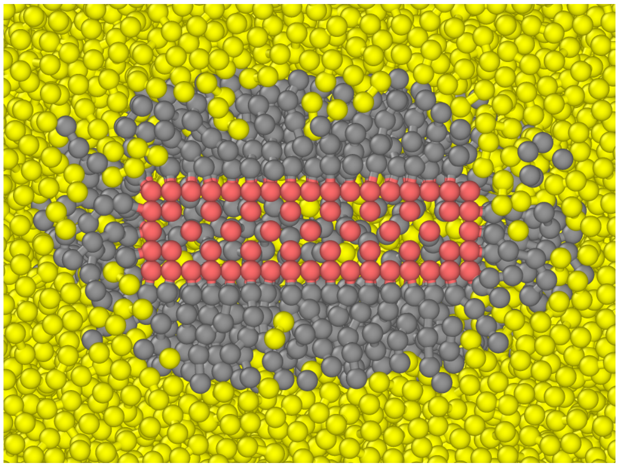

(c) 5 MARTINI beads

Figure S13: Cross section snapshots of nanoplatelets in octane with different ligand lengths. The platelets have 35.6 nm^2 base facet area and 1.5 nm thickness. CdSe beads are shown in red, ligand beads in grey, and solvent beads in yellow.

S1.7 Ligand grafting density and lattice constant

Nanocrystals are typically covered by a ligand shell.[12] In the case of nanoplatelets, for example consisting of CdSe, the ligand shell can be very dense.[11] In Figure S14 we compare the effect of different ligand grafting densities on the platelet face to face interaction. In our system setup the ligand grafting density is connected to the lattice constant, since the ligands are covalently bound to the surface beads/atoms. The larger the chosen lattice constant is, the smaller is the ligand grafting density. We choose the lattice constants in that way, that they result in the same base facet areas (27.3 nm^2). The thickness of the platelets change, dependent on the lattice constant (Table S3).

With decreasing ligand grafting density, the extrema shift to smaller surface-surface distances. This happens due to increased space available to the ligands and increased interdigitation of the ligands of the two interacting facets. The ligand grafting density also influences the strength of the interaction. We relate this to the resulting ligand packing density (Figure S16). At a high packing density the polymer brush becomes “hard”. This “hard” surface supports layer formation of the solvent and therefore leads to stronger solvation forces. On the other hand, a lower packing density results in a soft brush surface. As motivated in the main manuscript, such a soft ligand shell reduces solvent layer formation and the solvation forces.

The overall shape of the free energy curves is independent of the ligand grafting density, and we therefore expect that general trends for other parameters should be widely independent of the grafting density.

Table S3: Connection between ligand grafting density, lattice constant and nanoplatelet thickness

ligand grafting density (ligands / nm^2)	lattice constant (nm)	thickness (nm)
4.2	0.65	1.3
3.6	0.75	1.5
2.7	0.87	1.7

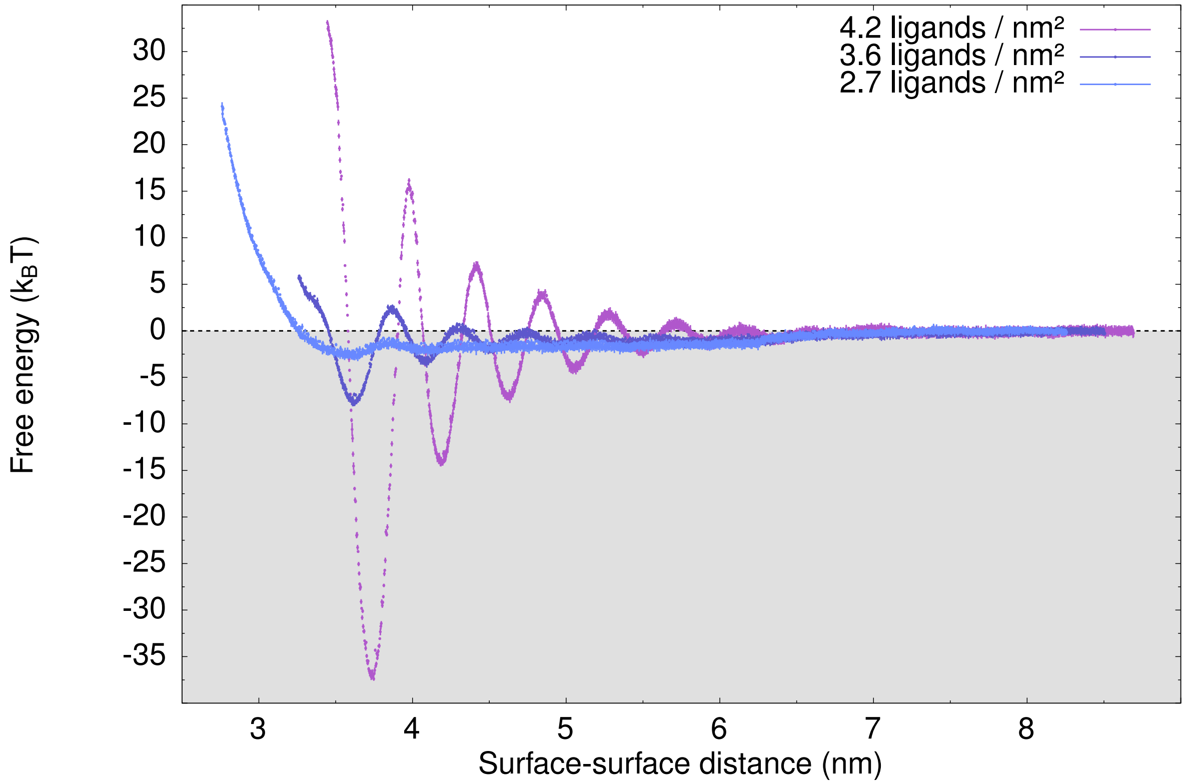

Figure S14: Effect of the ligand grafting density. Free energy curves of base facet to base facet interaction (error bars included). The platelets have 27.3 nm^2 base facet area. The platelet thickness changes depending on the ligand grafting density / lattice constant (Table S3).

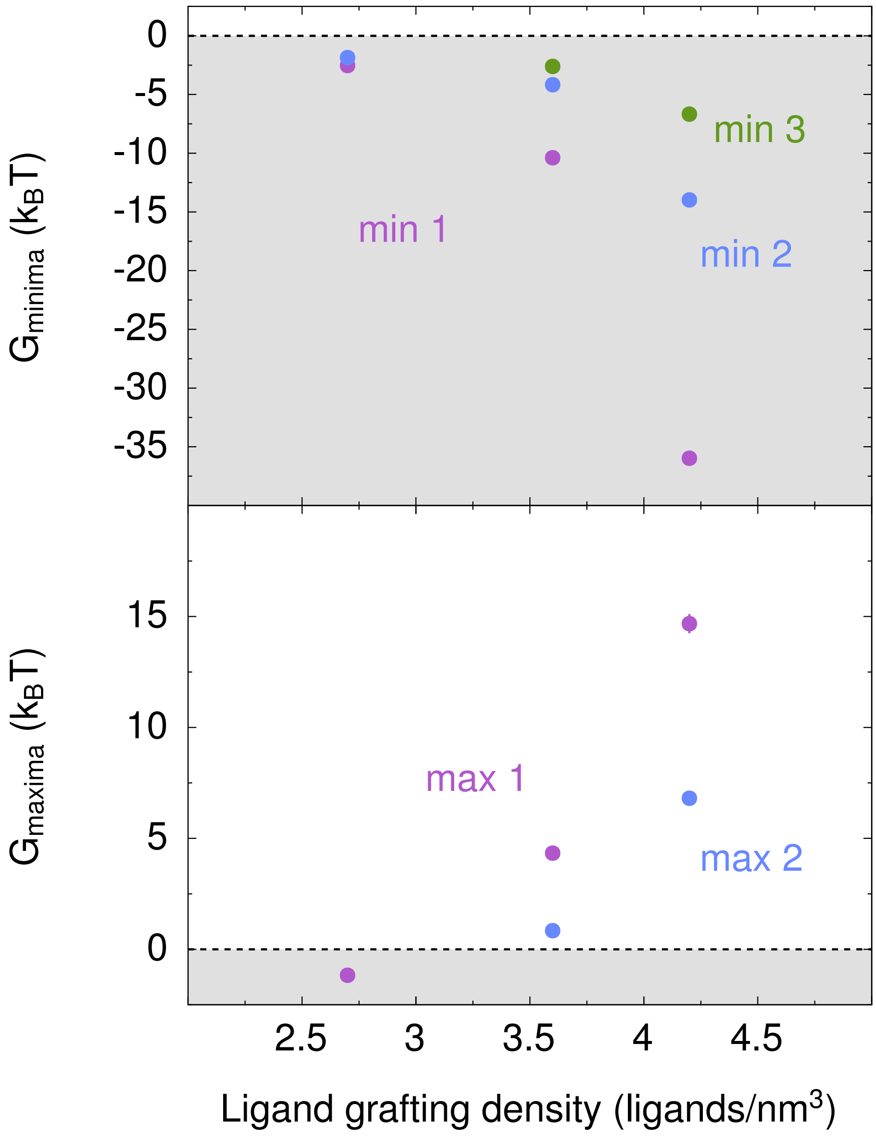

Figure S15: The variation of the ligand grafting density leads to a change in the strength of the interaction. Plotted are the depth of the free energy minima (panel **a**), and the height of the free energy maxima (panel **b**). Some error-bars of the fits are smaller than the points. We could not define a third minima and maxima in the free energy curve of the system with 2.7 ligands/nm^2.

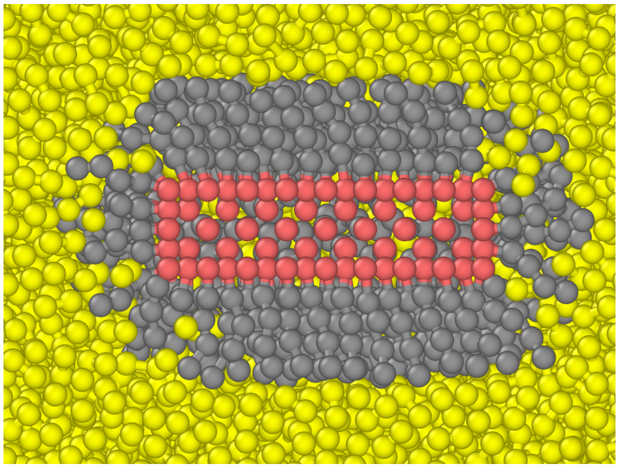

(a) 4.2 ligands/nm^2

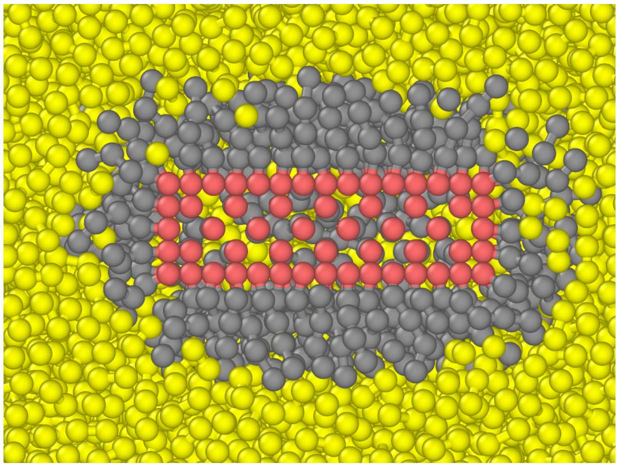

(b) 3.6 ligands/nm^2

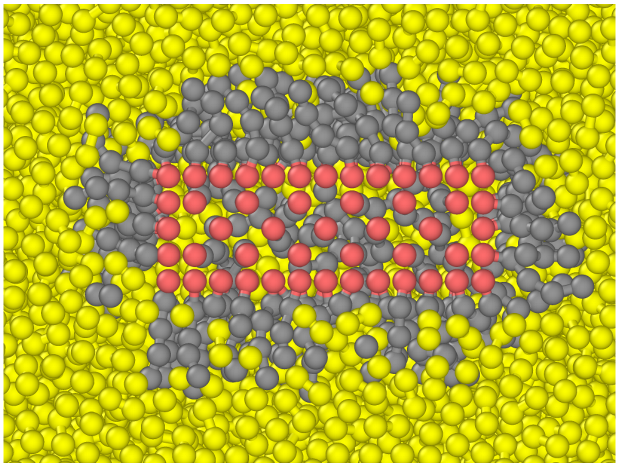

(c) 2.7 ligands/nm^2

Figure S16: Cross section snapshots of nanoplatelets in octane with different ligand grafting densities. The platelets have 27.3 nm^2 base facet area. The platelet thickness changes depending on the ligand grafting density / lattice constant (Table S3). CdSe beads are shown in red, ligand beads in grey, and solvent beads in yellow.

S1.8 Nanoplatelet thickness

We investigate the effect of the platelet thickness on the interaction between two platelet base facets. Here we compare a thickness of 1.5 nm^2 and 3.0 nm^2 (Figure S17). We find, that compared to other effects, like the platelet facet area, the platelet thickness has a minor effect on the interaction. In our simulation, the interaction gets slightly more attractive for a thicker platelet. This can be explained by the larger number of ligands on the side facets. They take up more space. Therefore, the ligand density increases at the edges of the base facets. The ligand shell becomes "harder".

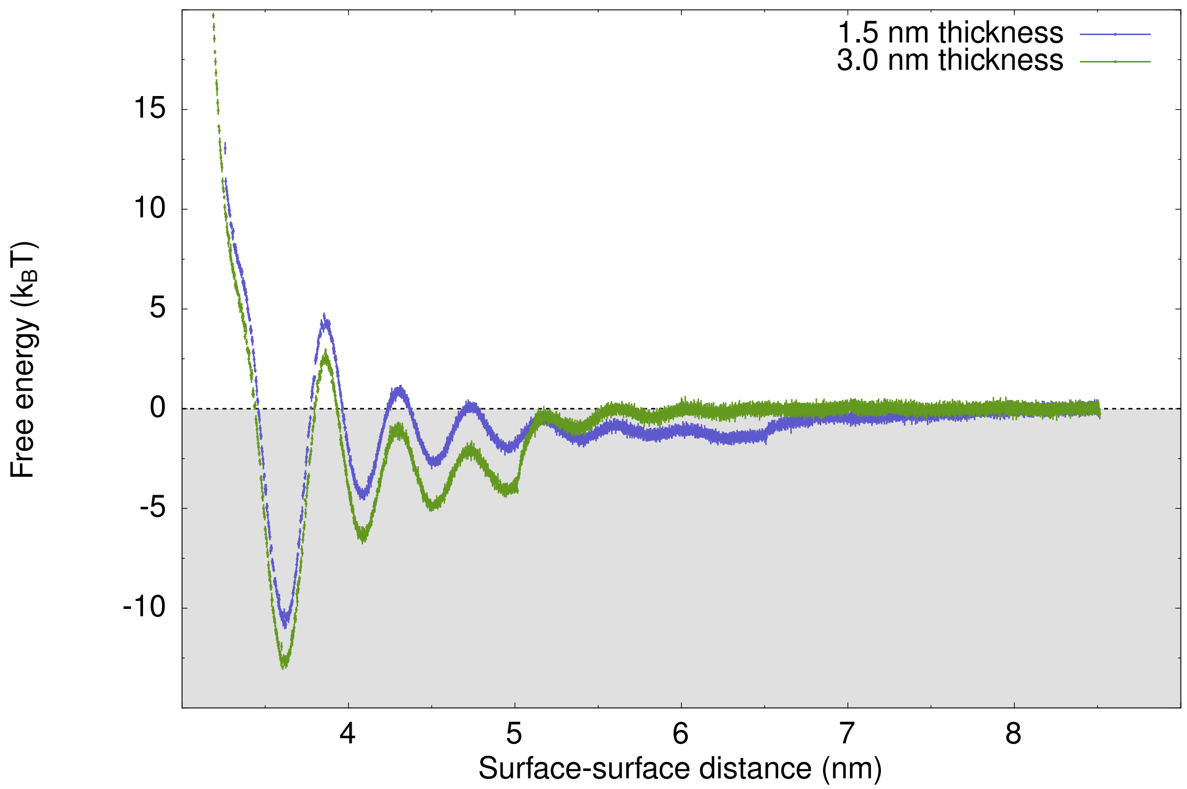

Figure S17: Effect of nanoplatelet thickness. Free energy curves of base facet to base facet interaction (error bars included). The platelets have 35.6 nm^2 base facet area. The thickness is varied between 1.5 nm and 3.0 nm.

S1.9 Nanoplatelet facet area

We run a number of simulations where we vary the platelet base facet area, while we keep the thickness constant at 1.5 nm (Figure S18). To specify the zero point of the free energy, we average the free energy between 9.75 nm and 10 nm. We assume that at this point the free energy is equal to that at infinite separation of the platelets. The solvation forces and layer formation become so small with a small base facet area (2.2 nm^2 and 5.0 nm^2) that the position of the minima/maxima can not be determined exactly. Therefore, the average position of the minima/maxima at larger platelets is chosen to determine the the free energy values (Figure 4).

The properties of the ligand shell depends on the platelet size (Figures S19 and S20). We find, that the interaction between the platelets is influenced by the shape and "softness" of the ligand shell (Figure 4).

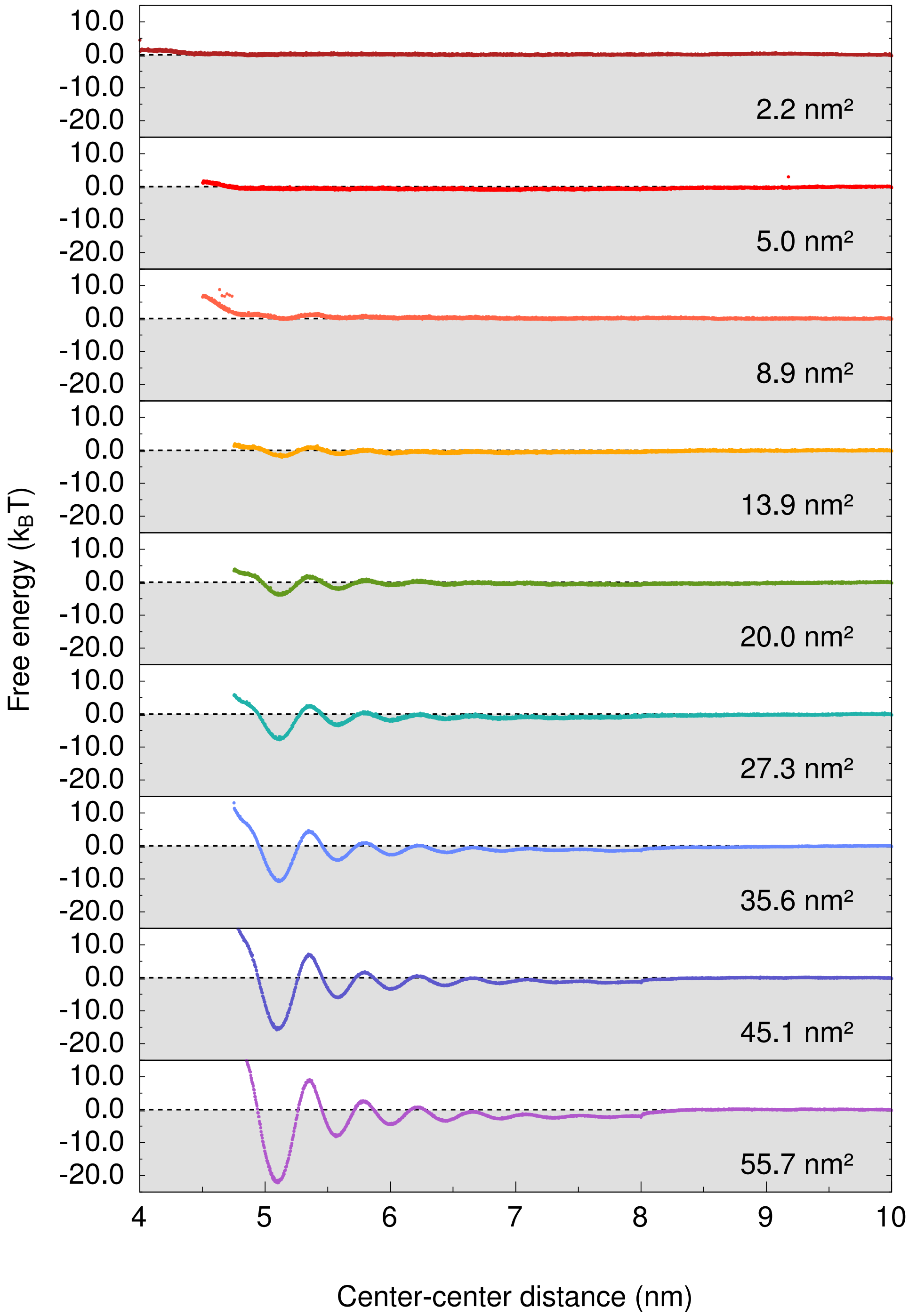

Figure S18: Free energy curves describing the interaction between sets of ligand coated nanoplatelets with different base facet areas (error bars included). The thickness of all platelets is 1.5 nm.

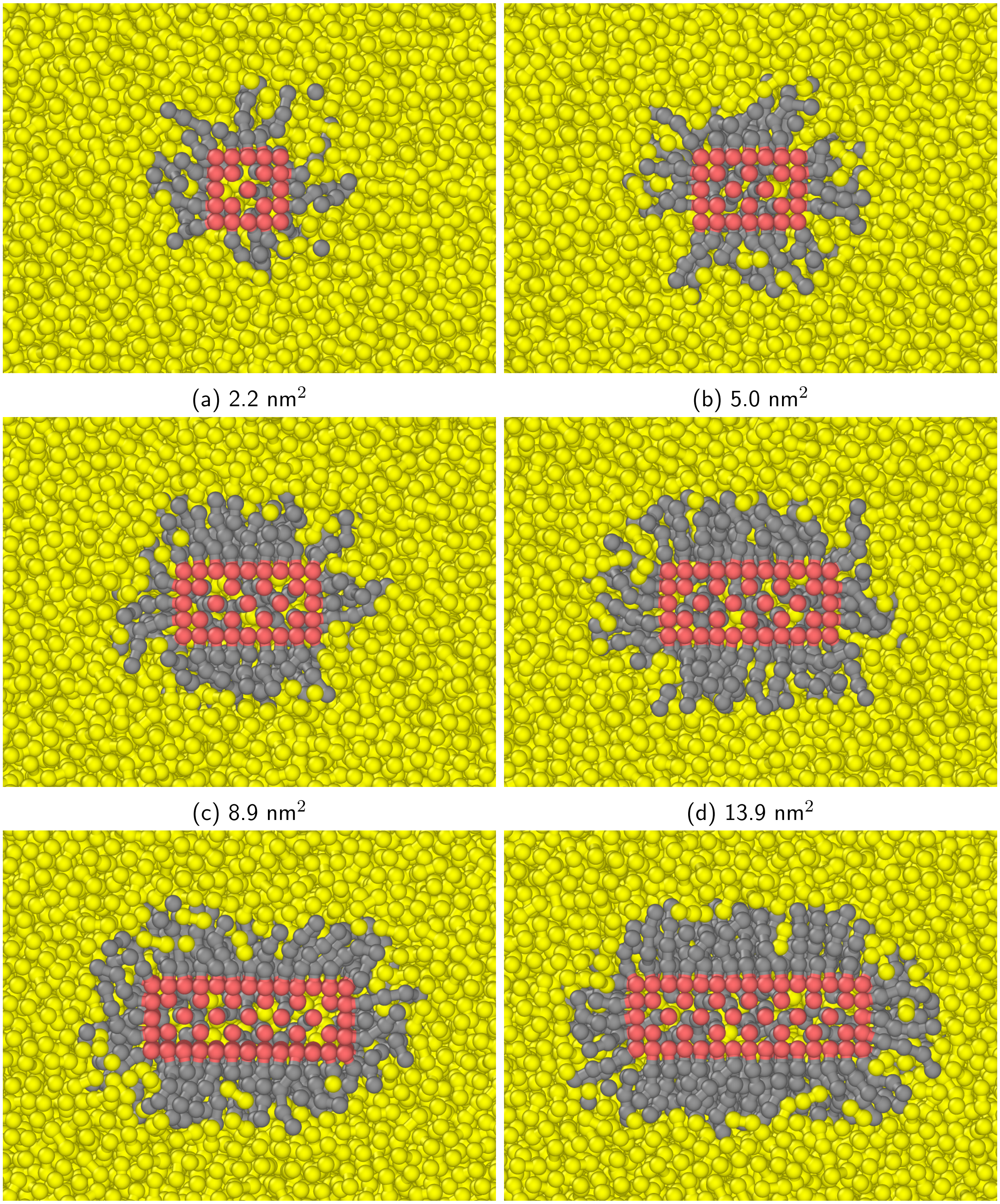

(a) 2.2 nm^2 (b) 5.0 nm^2 (c) 8.9 nm^2 (d) 13.9 nm^2 (e) 20.0 nm^2 (f) 27.3 nm^2

Figure S19: Cross section snapshots of platelets with different base facet areas. The thickness of the platelets is 1.5 nm. CdSe beads are shown in red, ligand beads in grey, and solvent beads in yellow.

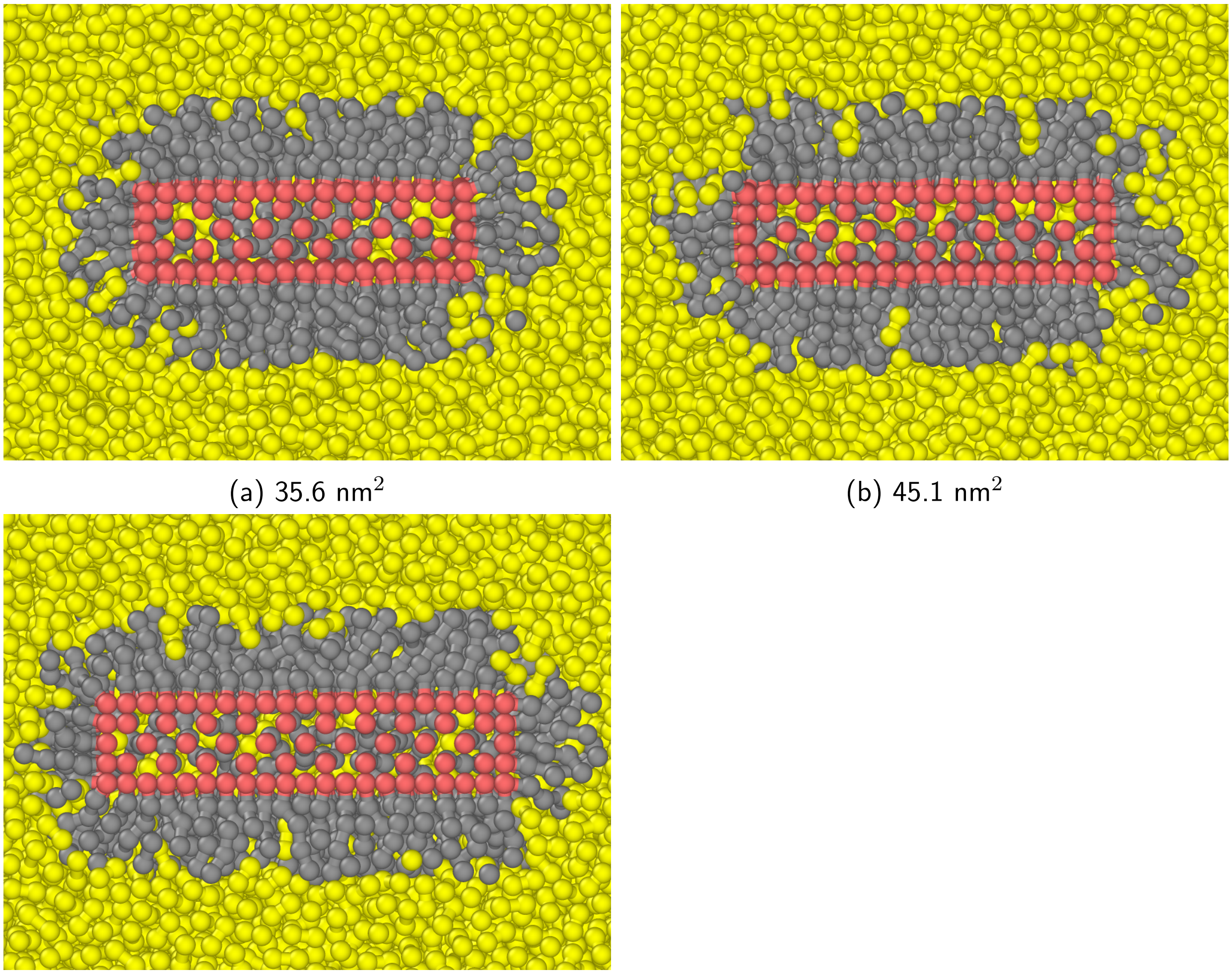

(a) 35.6 nm^2

(b) 45.1 nm^2

(c) 55.7 nm^2

Figure S20: Cross section snapshots of platelets with different base facet areas. The thickness of the platelets is 1.5 nm. CdSe beads are shown in red, ligand beads in grey, and solvent beads in yellow.

S1.10 Orientation Dependence of Nanoplatelet Interactions

In experiments CdSe nanoplatelets show the tendency to self-assemble into stacks.[4] To understand the nanoplatelet assembly process, we have to take into account the anisotropic shape of the nanoplatelets. The pair interaction depends on the relative orientation of the platelets. For example, the interaction is much weaker in the side facet to side facet orientation. For a platelet with a 35.6 nm^2 base facet area we find in this orientation only a shallow minimum and no barrier (see Fig. S21).

To understand why nanoplatelets form stacks, it is helpful to compare our CdSe system with another system that is similar at first glance. Balankura *et al.* studied triangular Ag nanoplates in an ethylene glycol solvent.[13] In their setup polyvinylpyrrolidone (PVP) molecules form a protective self-assembled monolayer on the nanoplates surfaces. Experiments have shown, that the triangular Ag nanoplates can aggregate laterally, "side-to-side" via an oriented attachment mechanism to create larger 2D sheets in ethylene glycol or N,N-dimethylformamide solvent.[14,15] Similar to the CdSe nanoplatelet system, Balankura *et al.* find in their calculated free energy curves a strong minimum in the base facet to base facet orientation and a weaker metastable minimum in the side facet to side facet orientation. The base facet to base facet attachment is favored thermodynamically. However, in both cases they also find a barrier. The barrier in the base facet to base facet orientation is significantly higher than in the side facet to side facet agglomeration. Therefore, they conclude that side to side attachment is kinetically favored .

The comparison with our current work on CdSe nanoplatelets raises the question of why the CdSe system behaves differently such that the agglomeration in base facet to base facet orientation is preferred.

The systems differ in various factors. The first difference that matters might be the different scaling of particle dimensions between experiments and simulations. Balankura *et al.* chose platelets with a much smaller size for in their simulations than found in experiment. In experiment the triangular Ag nanoplates have edge lengths ranging from 300–1000 nm

and thicknesses between 25–30 nm. However, simulations where done for plates with 2.12 nm thickness and an edge length of 8.09 nm. The area scaling factor for the side facets lies between 400 and 1750, the area scaling factor for the base facets between 1300 and 15000. It can be assumed that the strength of the interaction also scales with the area. If there is even a weak minimum in the free energy curve for the side to side attachment, it can be assumed that this is sufficient to enable a stable attraction / bond between the Ag plates.

In the case of CdSe nanoplatelets, side lengths vary between 5-20 nm and a typical thickness is 1.2 nm. This means that our simulations already cover typical platelet sizes. However, Jana *et al.* used slightly larger ones than we have calculated.[4] Here we get a scaling factor of less than 20 for the side facets, and a scaling factor of around 4 for the base facets. In Figure 4 of the main manuscript we have already discussed that the attraction between the CdSe nanplatelets increases with size. However, since the scaling factors are much smaller, it can be assumed that the attraction does not increase with size as much as in the case of the Ag plates. In view of the fact that we find only a weak minimum for the side to side orientation in our calculated free energy curve (Figure S21), it can be assumed that it will not be sufficient to enable agglomeration in the side-to-side orientation even with larger side lengths.

The question remains whether the barriers can prevent agglomeration in the base facet to base facet orientation. Here, we have to take into account the anisotropic shape of the CdSe nanoplatelets. Platelets can approach in different orientations. Due to high computational costs, we can not calculate the full free energy surface for the pair interaction. To show that there are assembling paths with much lower barriers, we therefore calculate one example free energy curve. We calculate the parallel side shift of two platelets (Figure S22). Thereby, we keep the center-center distance in face to face direction constant at 5.11 nm. This distance corresponds to the base facet to base facet first minimum (see Figure 3 in the main manuscript). Here, along this particular path, the barriers vanish.

These results show that the barrier in the base facet to base facet interaction is not

an insurmountable obstacle and that the pair interaction of the nanoplatelets is strongly dependent on the relative orientation. Therefore, we can conclude that free energy barriers obtained in the base facet to base facet interaction calculations are a higher bound, and more realistic assembly pathway where nanoplatelets can fully freely move and rotate will have lower free energy barriers.

At this point, the question may arise as to why the Ag system behaves differently. Could not the plates also slide sideways into the global minimum in the base facet to base facet orientation? This is not necessarily the case. A reason we can think of is the different cause of the barrier. PVP molecules which bind to the surface of the Ag plates have to move out of the way as two facets approach. They do this in two ways: they migrate to the neighboring surfaces of the aggregate, or they desorb from the aggregate in the solvent.[13]

Regardless of the direction of approach, PVP molecules must move. For an exact consideration we unfortunately lack inside into the free energy here. On the basis of the information available, we would assume that there is a barrier regardless of the approach direction. Due to the area and the different number of PVP molecules to be moved, the barrier for agglomeration in the base facet to base facet orientation is likely to be greater than that for agglomeration in the side facet to side facet orientation.

Since in the case of the CdSe system the ligands and the solvent are chemically similar, this does not apply to the CdSe nanoplatelet system. Here, barriers arise due to the layer formation of the solvent. However, when approaching from the side or other directions, this effect largely disappears and the barriers disappear or are greatly reduced.

The comparison between these two systems illustrates how strongly the pair interaction depends on the interaction of the different forces and effects.

For the CdSe nanoplatelet system, we can conclude from this analysis that the shape of the CdSe nanoplatelets in interaction with the solvation forces leads to a pair interaction that depends on the relative orientation. Depending on the relative orientation, the barriers are so small that they can be overcome.

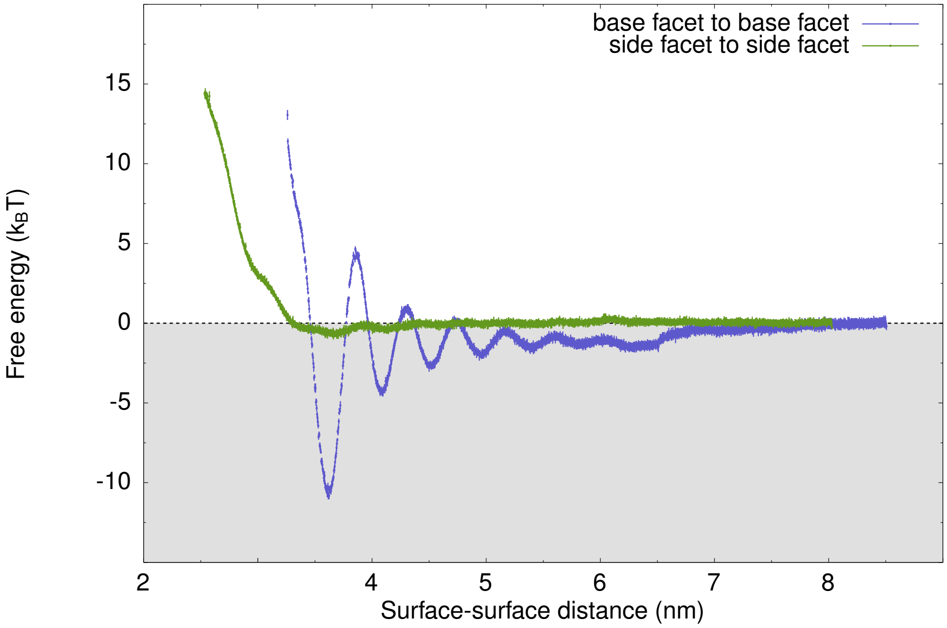

(a)

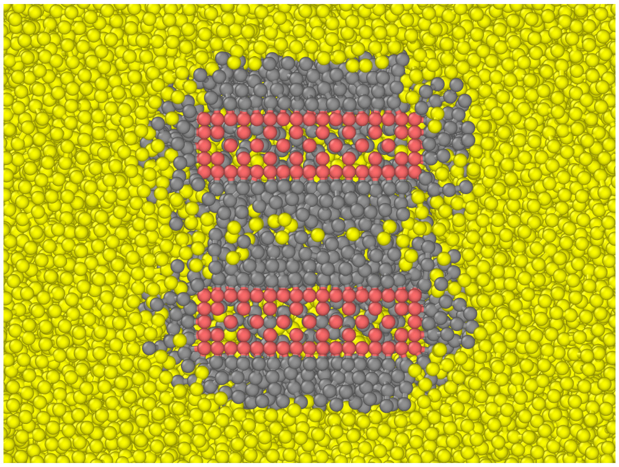

(b) base facet to base facet interaction

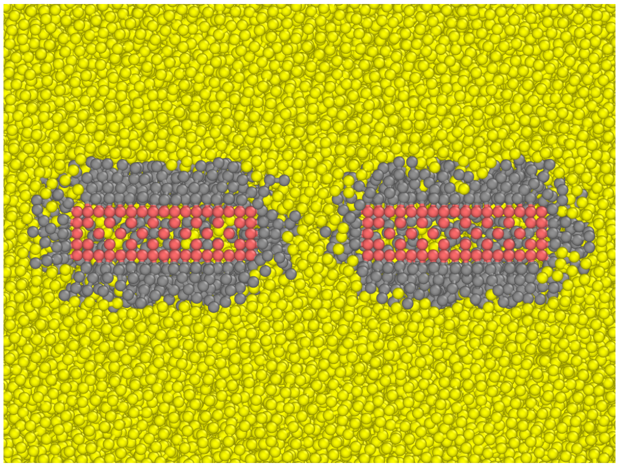

(c) side facet to side facet interaction

Figure S21: Comparison of the base facet to base facet interaction and the side facet to side facet interaction. The platelet base facet area is 35.6 nm^2, and the thickness 1.5 nm. The side facet area is 8.9 nm^2. Panel **a** includes the free energy curves. A snapshot of the base facet to base facet orientation is shown in panel **b**, and a snapshot of the side facet to side facet orientation in panel **c**.

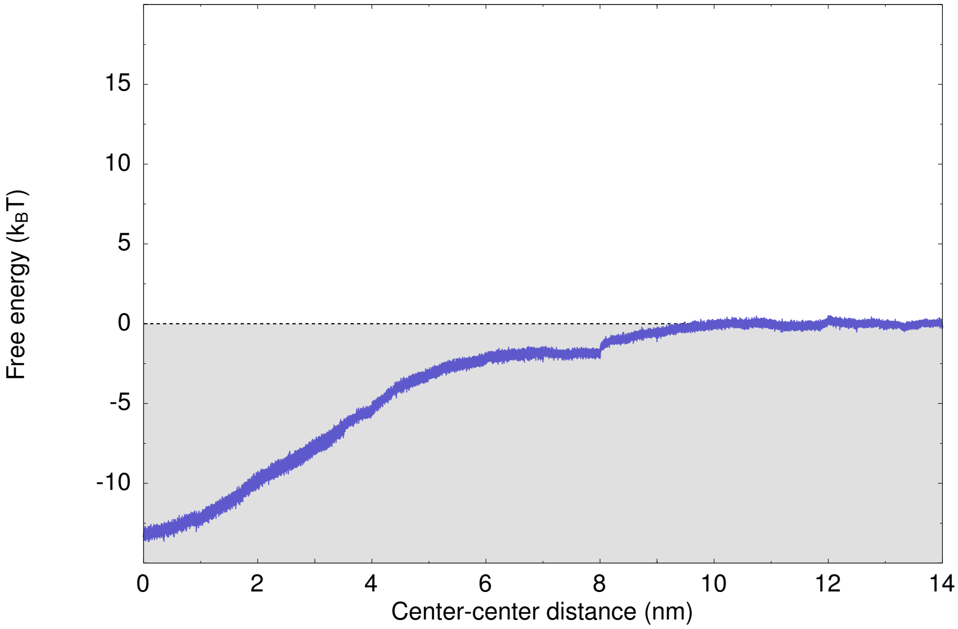

(a)

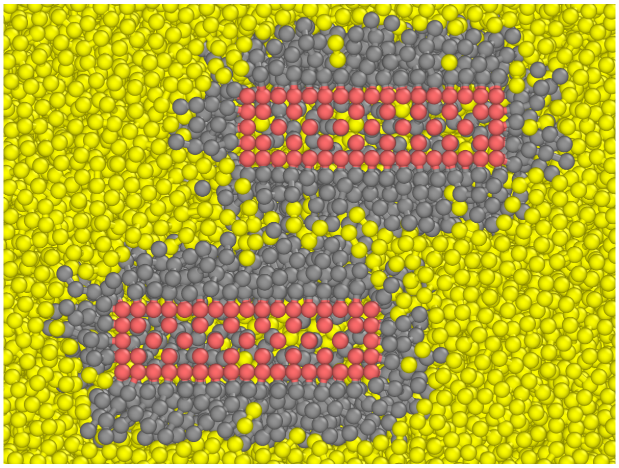

(b) 3.0 nm distance

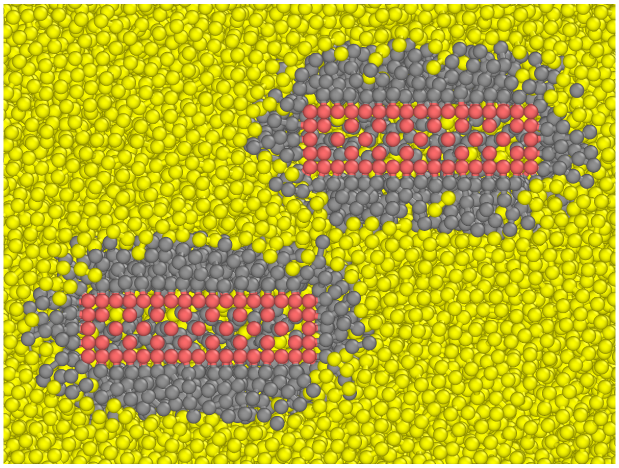

(c) 6.0 nm distance

Figure S22: Free energy curve describing the interation on a parallel side shift of two nanoplatelets (panel **a**). The platelet base facet area is 35.6 nm^2, and the thickness 1.5 nm. The side facet area is 8.9 nm^2. Panel **b**, and panel **c** are showing simulation snapshots. In panel **b** the center-center distance is 3.0 nm, and in panel **c** it is 6.0 nm.

S2 Methods

We create a coarse grained model system to describe nanoplatelets in octane solvent (Figure 1 in the main manuscript). A coarse grained approach is well suited for identifying qualitative trends. Due to the reduction in particle numbers, it requires dramatically less computational power than all atom simulations. For our model system we use the well-studied CdSe nanoplatelets as a reference system. However, our setup is very general and resulting trends should describe also the behaviour of other similar platelet systems.

S2.1 Model System Setup

We use the the MARTINI force field to describe the interaction between different coarse grained beads.[7] This force field is optimized to describe the liquid state of organic molecules and it does not include an explicitly representation for the solid state or of nanoparticles. Therefore, we do not describe the inner atomic structure of the platelets, but model the nanoplatelets by surface beads, which we use as grafting points for their ligands. CdSe nanoplatelets typically show a rectangular shape.[4,16,17] Here, we model platelets with square base facets. As on CdSe nanoplatelets,[9,10] we use a face centered surface lattice. To avoid solvent and ligand molecules entering the inside of a nanoplatelet, we apply a repelling 12-6 Lennard-Jones potential to interactions between the solvent / ligand beads and the surface beads ($\epsilon = 2.0$ kJ/mol, $\sigma = 0.62$ nm).

One important parameter is the ligand grafting density,[18] which is proportional to the distance between two grafting points and therefore to the lattice constant. We choose the distance between two ligand grafting sites such that two neighbouring ligands are in the minimum of the interacting 12-6 Lennard-Jones potential. Therefore, we choose a lattice constant of 0.746 nm, ending up with 3.6 ligands per nm^2 (see Section S2.2 for further details).

To calculate the mass of each platelet, we employ the mass density of CdSe ρ= 5.7 g/cm^3.

The core-core van der Waals interaction is not taken into account in our model. It is largely screened by the ligand shell and the surrounding solvent (see Section S1.1 for discussion).

In our setup, aliphatic lipid tails are bound covalently as ligands to the surface. The MARTINI force field is using a 4 to 1 mapping. Four CH_x groups of a ligand or solvent molecule are described in our model by one C1 MARTINI bead ($\epsilon = 3.5$ kJ/mol, $\sigma = 0.47$ nm). If not otherwise stated, 4 beads are used to describe the ligand molecules. This corresponds to an aliphatic lipid tail with a length of 16 carbon atoms. We use octane solvent as represented by two MARTINI beads. To simplify the system setup we utilise the HOOBAS molecular builder tool.[19]

S2.2 Choice of ligand grafting density for the simulations

Assuming that the ligands graft to the Cd surface atoms and a lattice constant of 0.608 nm, the maximum density of ligands grafting to the CdSe nanoplatelet surface would be around 5.4/nm^2. Experimental measured densities are close to this value.[10,11]

In our simulations, we use a smaller value. We do this because the ligand density in the simulations depends on the bead size, and C1 MARTINI are relatively large ($\sigma = 0.47$ nm). For example, the bead size in the united atom TraPPE force field lies between $\sigma = 0.385$ nm and 0.395 nm.[8] In our test simulations we find, that a ligand grafting density higher than 4.2 ligands / nm^2 leads to deformation of the ligand shell. Some surface-ligand bonds are overstretched or strongly compressed. Artifacts occur at the ligand shell surface.

Our analysis in Section S1.7 shows that while the strength of the interaction depends on the ligand grafting density, the overall shape of the free energy curves is independent of the ligand grafting density. We therefore expect that general trends for other parameters should be largely independent of the grafting density.

For our model system we choose a lattice constant of 0.746 nm, ending up with 3.6 ligands per nm^2. At this lattice constant, the distance between two neighboring grafting sites match the distance in which the interacting 12-6 Lennard-Jones potential of the ligands has its

minimum (see Figure S23).

In Section S1.2, we compare our setup with the MARTINI force field to a united atom setup with the TraPPE-UA force field. There, we discuss the solvent layering at the ligand-solvent interface of infinite large platelets. Both systems describe a ligand shell, which on one side is dense and forms a protective shell around the nanoplatelets and on the other side is soft enough that solvent molecules still can mix with it at its interface. We conclude in Section S1.2 that our setup with the MARTINI model and the chosen lattice constant is qualitatively an appropriate description of the pair interaction, while there will be quantitative differences in the strength of the interaction.

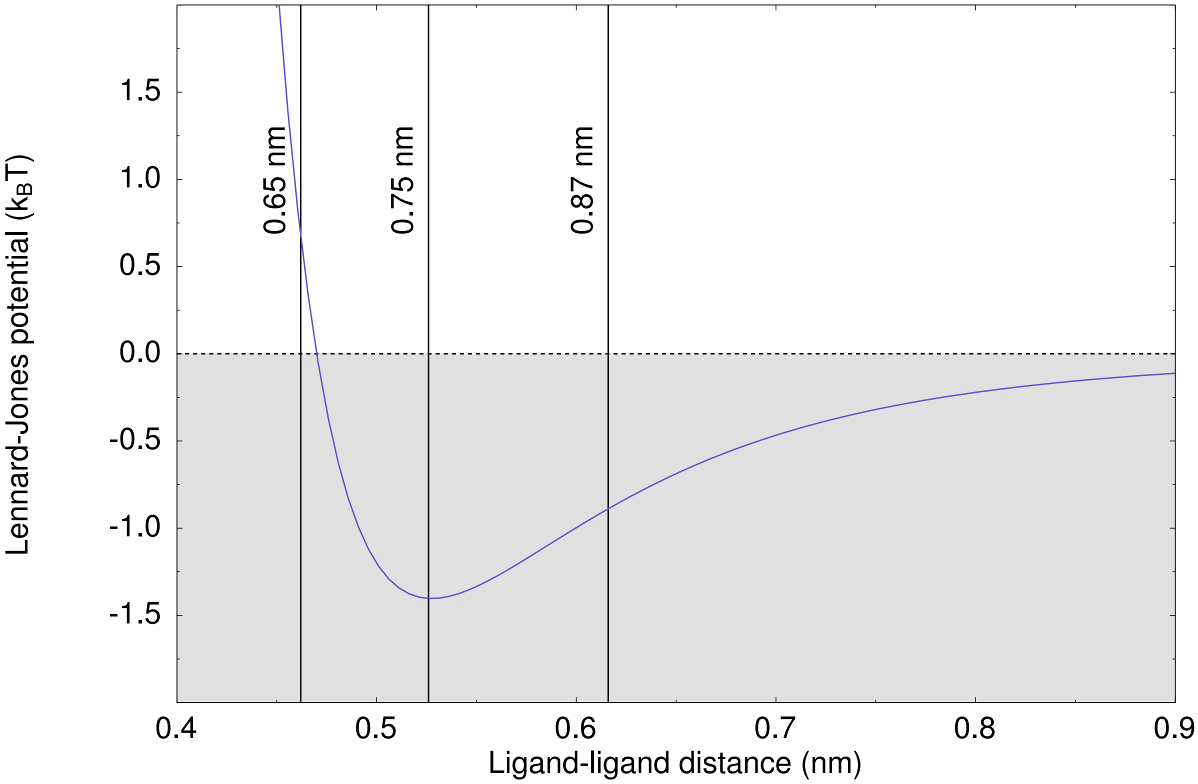

Figure S23: Ligand grafting distances, dependent on the lattice constants (labels), in relation to the 12-6 Lennard-Jones potential acting between ligand beads.

S2.3 Molecular Dynamics Simulations

We use the HOOMD package (v2.6.0)[20–22] for our molecular dynamics simulations. Our simulations are performed at a constant temperature of 300 K and a constant pressure of 1 atm (NPT) in cubic, periodic simulation boxes. For the barostat and thermostat, we employ the standard HOOMD NPT integrator, based on the Martyna-Tobias-Klein equations of motion.[23–25] We use a time step of 0.02 ps and the barostat-thermostat parameters tauP=1 and tau=1.

In coarse grained simulations the interpretation of time scales is not straightforward. The main reason is that the underlying energy landscape is smoother than in an all atomistic simulation and the dynamics are faster. We use an effective time which is four times the simulation time.[7] We use OVITO[26] to analyze the simulations and take snapshot images.

S2.4 Free Energy Calculations

The free energy can give an insight in interaction mechanics. We calculate the free energy for the base facet to base facet interaction in z direction of two nanoplatelets (see Figure 1 in the main manuscript) using multiple windows umbrella sampling simulations.[27] We post process the umbrella sampling simulations using Weighted Histogram Analysis Method (WHAM).[27–29] The WHAM analysis is performed using the tool of D. Bauer.[30]

Rotation of platelet objects is avoided by disabling anisotropic integration in HOOMD. This affects only the platelets. Platelets are constrained to prevent a sideways movement of the platelets by built-in HOOMD commands. We further enforce by a custom plugin that the x and y positions as well as velocities are set to zero to avoid numerical noise. Only movements of the two platelets toward or away from each other in z direction are allowed.

A harmonic umbrella potential is applied between the centers of the two nanoplatelets:

$$U_{pot} = \frac{1}{2}\kappa \left(r - r_0\right)^2 , \tag{S4}$$

where we use a force constant of $\kappa = 50000$ kJ/mol. r is the distance in z and r_0 is the preferred distance targeted by the umbrella potential.

Before we start a new umbrella simulation, we set up a start system in which the two nanoplatelets have a small distance from each other (at 4 ligand beads ≤ 4.5 nm). This start system is relaxed to 300 K and 1 atm. We change r_0 by steps of $\Delta r_0 = 0.005$ nm or $\Delta r_0 = 0.0025$ nm, dependent on the platelet size / simulation box size (see Tables S7-S11). An umbrella simulation going from 4.75 nm to 10.0 nm platelet distance includes 1050 windows. These windows we divide into intervals of two or more windows.

If a window is at the beginning of the interval, we start the simulation from the start system, otherwise we start the new simulation with the result of the previous window. If we start from the start system and $r_0 \leq 5.5$ nm we equilibrate the system with the NPT integrator by slowly changing r_0 in steps of 0.1 nm until it reaches the value of that window. In each step we let the system equilibrate for 1.6 ns and additionally at the end for 24 ns. Otherwise, if we start from the previous window, or if $r_0 \geq 5.5$ nm we equilibrate the system for 40 ns.

Then in a production run, we obtain 250 data points over an effective time interval of 40 ns. From the probability distribution for the center-center distances in each window, the free energy curve is calculated using WHAM (see next section for WHAM parameter).

In general we perform the umbrella simulations going from small to large distances. To validate the umbrella sampling simulations, we also perform independent umbrella sampling simulations going from large to small distances. The differences between the results are negligible (Figure S24).

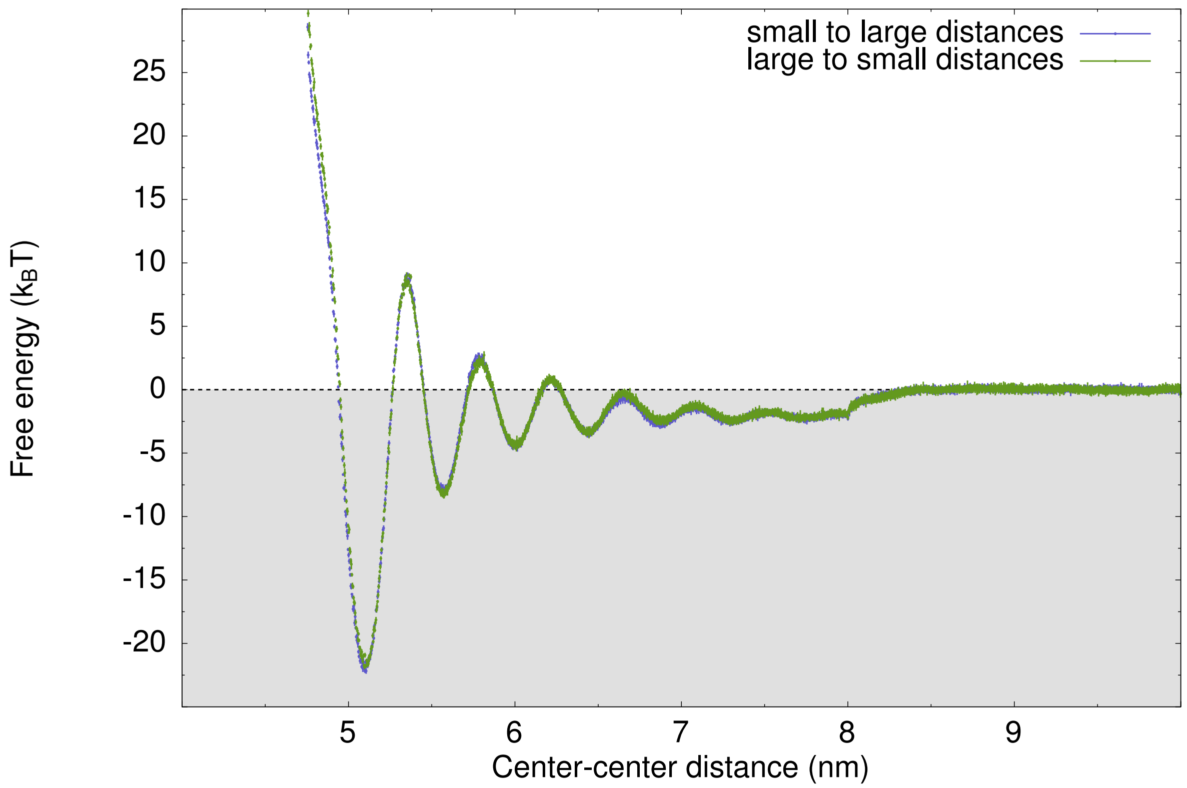

Figure S24: Example for test of the directionality of the umbrella simulations. The two nanoplatelets have facets with 55.7 nm^2. One umbrella calculation is done going from small to large distances, one is done going from large to small distances.

S2.5 WHAM parameter

We use the WHAM code of Daniel Bauer in the version 1.0.0.[30] Important parameters to choose are the tolerance and the number of bins. The error of WHAM calculations is estimated by bootstrapping. In Figures S25-S27 examples for our parameter tests are plotted. In Table S4 we list the parameters we finally use to calculate free energy curves with WHAM.

Table S4: Parameters used to calculate the free energy curves employing WHAM.

Number of bins	Tolerance	Bootstrapping steps (bt)
305 bins / nm	1e-06 kJ/mol	10

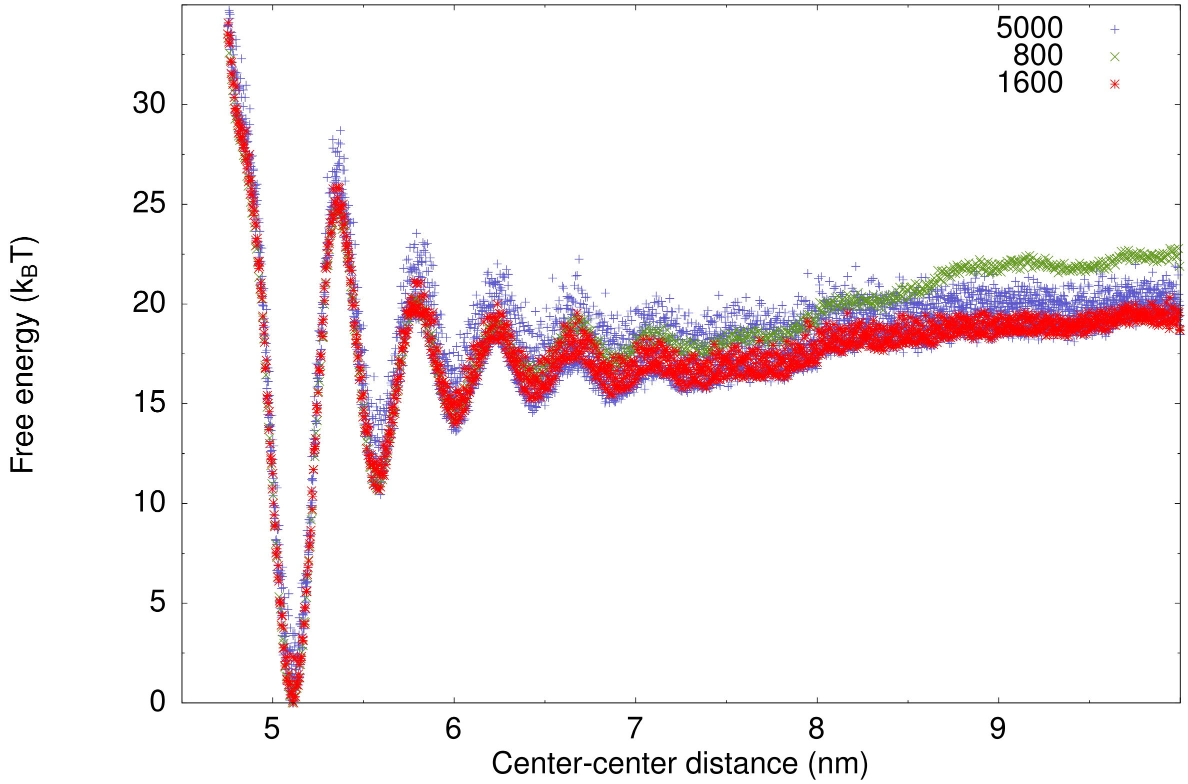

Figure S25: Test WHAM parameter number of bins (27.3 nm^2, 100k solvent molecules): Comparison of different bin numbers. A too high value leads to a high fluctuation, a too low to a shift in the free energy. We choose 1600 bins (305 bins/nm) for all WHAM calculations.

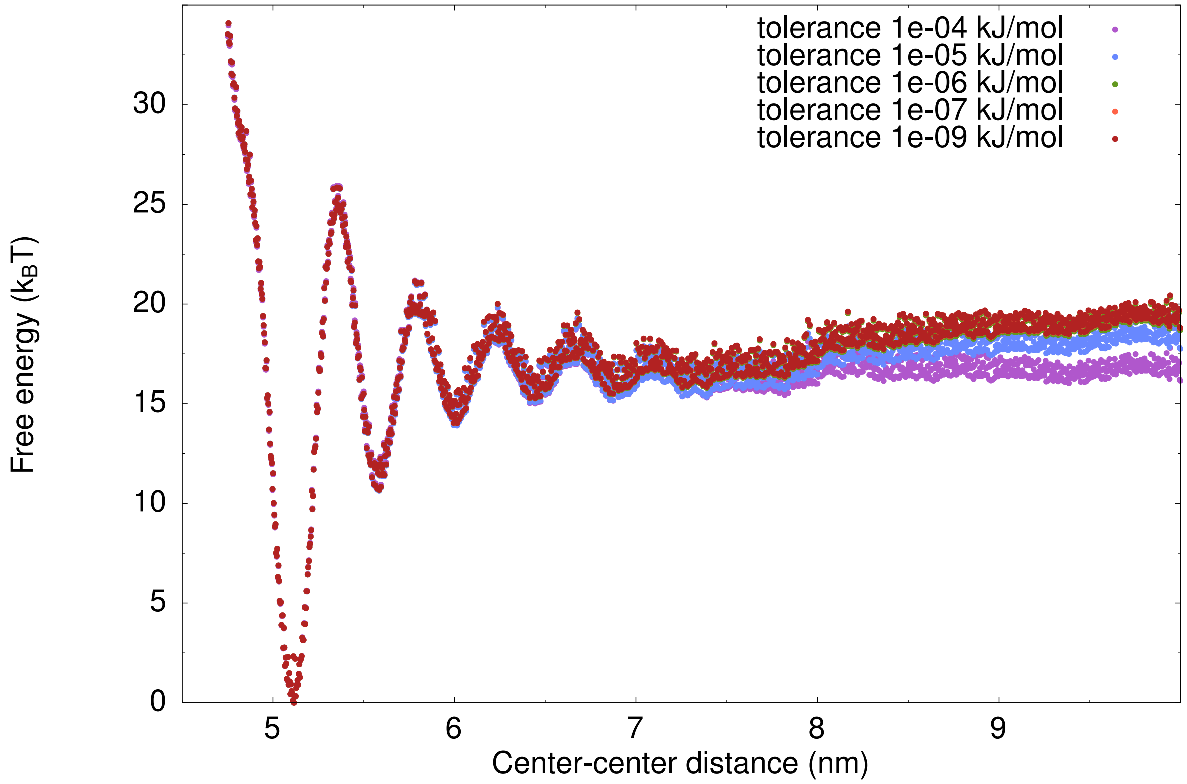

Figure S26: Test WHAM parameter tolerance (27.3 nm^2, 100k solvent molecules): Comparison of different values for the tolerance. For tolerances smaller than 1e-06 kJ/mol changes in the free energy are quite small (Green points of 1e-06 kJ/mol are mainly directly below orange points of 1e-07 kJ/mol and the red points of 1e-09 kJ/mol.). We choose a tolerance of 1e-06 kJ/mol for our WHAM calculations.

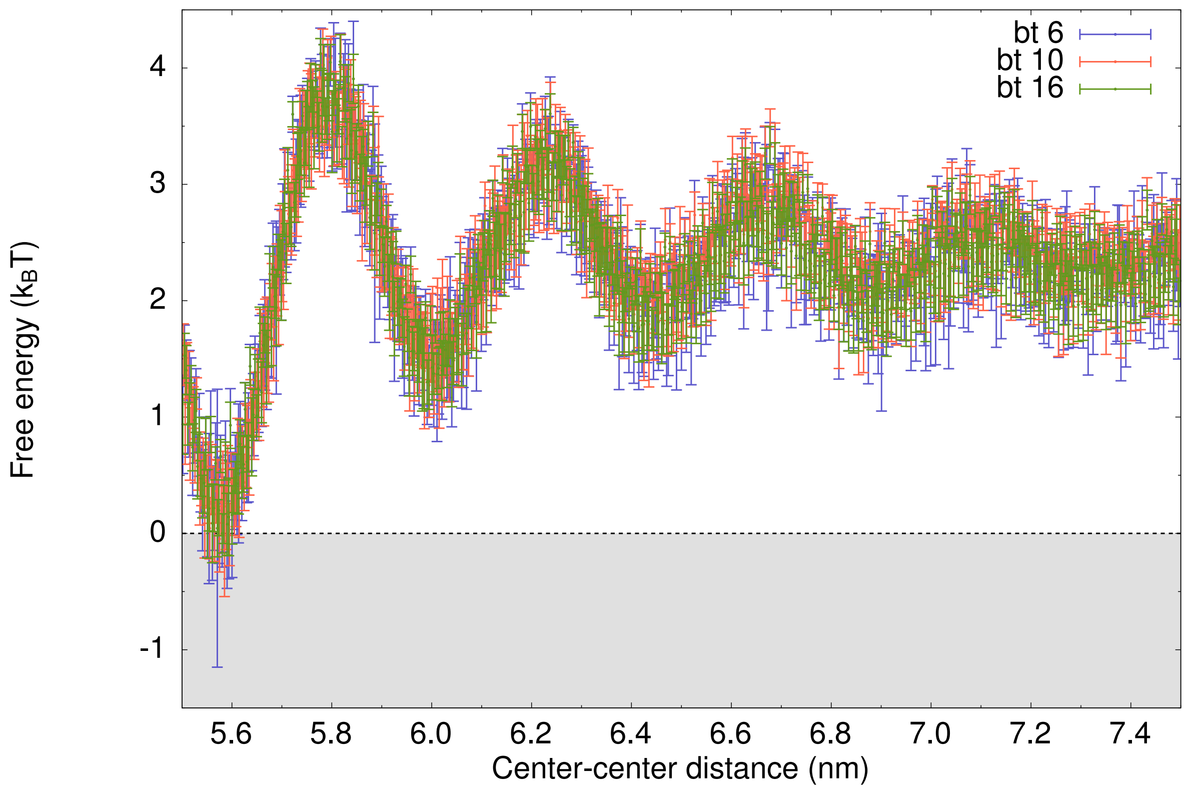

Figure S27: Comparison of different numbers (bt) of bootstrapping runs (27.3 nm^2, 100k solvent molecules). The differences in the errors are small. We use 10 bootstrapping runs to evaluate the error of free energy curves.

S2.6 Base facet area, solvent number and average box size

The number of solvent molecules and therefore the box size is varied dependent on the nanoplatelet size, the number of platelets and the solvent length. If the simulation box is too small, the free energy deviates. In this section we list for all simulations the base facet area, the number of solvent molecules and the resulting average simulation box side length. In the case of the Umbrella simulations which are used to calculate the free energy curves, the average side length is determined at a window with approximate 10 nm center-center distance between the platelets (11.5 nm in the case of 3.0 nm platelet thickness).

Table S5: Simulation parameters we use to run the unconstrained simulations with two platelets.

Base facet area (nm^2)	# of solvent molecules	Average simulation box side length (nm)
20.0	100k	28.5
27.3	100k	28.5
35.6	100k	28.6
45.1	100k	28.6
55.7	350k	43.2

Table S6: Simulation parameters we use to run the unconstrained simulations with three platelets.

Base facet area (nm^2)	# of solvent molecules	Average simulation box side length (nm)
20.0	150k	32.6
45.1	250k	38.7

Table S7: Simulation parameters we use in the analysis of the effect of the ligand shell.

	Base facet area (nm^2)	# of solvent molecules	Average simulation box side length (nm)	Δr_0 (nm)
without ligand shell	35.6	100k	28.5	0.0025
with ligand shell	35.6	250k	28.6	0.0025

Table S8: Simulation parameters we use in the analysis of the effect of the ligand length.

Ligand length	Base facet area (nm^2)	# of solvent molecules	Average simulation box side length (nm)	Δr_0 (nm)
3	35.6	100k	28.5	0.005
4	35.6	250k	28.6	0.0025
5	35.6	100k	28.6	0.005

Table S9: Simulation parameters we use in the analysis of the effect of the ligand grafting density.

Ligand density	Base facet area (nm^2)	# of solvent molecules	Average simulation box side length (nm)	Δr_0 (nm)
2.7	27.3	100k	28.5	0.005
3.6	27.3	100k	28.5	0.005
4.2	27.3	100k	28.5	0.005

Table S10: Simulation parameters we use in the analysis of the effect of the platelet thickness.

Platelet thickness (nm)	Base facet area (nm^2)	# of solvent molecules	Average simulation box side length (nm)	Δr_0 (nm)
1.5	35.6	250k	38.6	0.0025
3.0	35.6	400k	45.1	0.0025

Table S11: Simulation parameters we use in the analysis of the effect of the platelet facet area.

Base facet area (nm^2)	# of solvent molecules	Average simulation box side length (nm)	Δr_0 (nm)
2.2	100k	28.4	0.005
5.0	100k	28.4	0.005
8.9	100k	28.4	0.005
13.9	100k	28.5	0.005
20.0	100k	28.5	0.005
27.3	100k	28.5	0.005
35.6	250k	38.6	0.0025
45.1	250k	38.6	0.0025
55.7	350k	43.2	0.0025

Table S12: Simulation parameters we use in the calculation of the free energy curve for the side to side interaction.

Base facet area (nm^2)	# of solvent molecules	Average simulation box side length (nm)	Δr_0 (nm)
35.6	250k	38.6	0.0025

Table S13: Simulation parameters we use in the calculation of the free energy curve for the parallel side shift interaction.

Base facet area (nm^2)	# of solvent molecules	Average simulation box side length (nm)	Δr_0 (nm)
35.6	250k	38.6	0.0025

S2.7 Simulation setup for infinitly large platelets

In Section S1.2, we study infinitly large platelets. We compare a setup with the MARTINI force field to a setup with the united atom TraPPE-UA force field. Figures S2b and c show snapshots of the setups. For both of these setups, we create two ligand grafted surfaces. We chose a periodic simulation box, which is commensurate with the platelet dimension. The barostat only adjusts the z dimension of the box, according to the zz component of the stress tensor. This setup therefore describes an infinitly large platelet.

The surface beads, which serve as grafting points for the ligands, are arranged in a face centered grid, as found on the surface of CdSe nanoplatelets.[9] In the TraPPE-UA setup, we use the CdSe lattice constant 0.608 nm, while we use 0.746 nm in the MARTINI setup. In the TraPPE-UA setup, we add additional surface beads in between, to prevent the solvent entering the middle of the platelet.

In the MARTINI setup, we describe the ligands by four C1 MARTINI beads, and the octane solvent molecules by two. The interaction parameters are given in Section S2.1.

In the TraPPE-UA setup, the ligands consist of 16 CH_x beads, corresponding to the four C1 MARTINI beads, and the solvent molecules are described by 8 CH_x beads. Beads in the middle of a molecule are CH_2 beads. The last bead of the ligand molecules, and the first and last bead of the solvent molecules are CH_3 beads.

We describe the interactions between the united atom beads by the TraPPE-UA force field,[8] extended by a bond stretching term.[31] Since the HOOMD version we use does not include analytical tail corrections of the Lennard-Jones interaction, we instead increase the cutoff to $r_{\text{cutoff}} = 1.8$ nm. To describe the interaction between the surface beads and the CH_x beads, we choose the parameters given in Table S14. These parameters actually describe the interaction of CdS with CH_x beads.[32] However, this should be a reasonable approximation.

In both setups, simulations are performed at a constant temperature of 300 K, and a constant pressure of 1 atm. To control the temperature, we use a Dissipative Particle Dynamics (DPD) thermostat.[33] For pressure control, we use a Martyna-Tobias-Klein barostat.[23]

In the MARTINI setup we use time steps of 0.02 ps, and in the TraPPE-UA setup time steps of 0.002 ps. Solvent and ligand densities are averaged over 5000 snapshots, in both cases. Simulation time in the TraPPE-UA setup is 200 ns, while it is 200 µs in the MARTINI setup.

Table S14: Interaction Parameter we use in the TraPPE-UA setup

	CH_3		CH_2		CdSe	
	ϵ in kJ/mol	σ in nm	ϵ in kJ/mol	σ in nm	ϵ in kJ/mol	σ in nm
CH_3	0.815	0.375	0.557	0.385	0.599	0.354
CH_2	0.557	0.385	0.382	0.395	0.466	0.354

S2.8 Data availability

Data supporting the results reported in this paper are openly available at Zenodo[34] (DOI: 10.5281/zenodo.7385599). Due to space limitations we cannot include all the simulation trajectories. Therefore, in this dataset, we include an example from one of the umbrella sampling simulation (35.6 $nm^2 \times 1.5$ nm, 4 ligand beads, 250k solvent molecules), and the input and output data of WHAM calculations to obtain the free energy curves. In addition, we include the simulation data from the comparison of the MARTINI and the TraPPE-UA force fields. Further data and simulations trajectories are available upon request from the authors.

References

(1) Rabani, E. An interatomic pair potential for cadmium selenide. *The Journal of Chemical Physics* **2001**, *116*, 258.

(2) Hough, D. B.; White, L. R. The calculation of hamaker constants from liftshitz theory with applications to wetting phenomena. *Advances in Colloid and Interface Science* **1980**, *14*, 3–41.

(3) Israelachvili, J. *Intermolecular and Surface Forces (3. Edition)*; Academic Press, 2011.

(4) Jana, S.; Phan, T. N.; Bouet, C.; Tessier, M. D.; Davidson, P.; Dubertret, B.; Abécassis, B. Stacking and Colloidal Stability of CdSe Nanoplatelets. *Langmuir* **2015**, *31*, 10532–10539.

(5) Napper, D. H. Steric Stabilization. *Journal of Colloid and Interface Science* **1977**, *58*, 390–407.

(6) Jiang, K.; Pinchuk, P. Temperature and size-dependent Hamaker constants for metal nanoparticles. *Nanotechnology* **2016**, *27*, 345710.

(7) Marrink, S. J.; Risselada, H. J.; Yefimov, S.; Tieleman, D. P.; Vries, A. H. D. The MARTINI force field: Coarse grained model for biomolecular simulations. *Journal of Physical Chemistry B* **2007**, *111*, 7812–7824.

(8) Martin, M. G.; Siepmann, J. I. Transferable Potentials for Phase Equilibria. 1. United-Atom Description of n-Alkanes. **1998**, *102*, 2569–2577.

(9) Ko, J. H.; Yoo, D.; Kim, Y. H. Atomic models for anionic ligand passivation of cation-rich surfaces of IV-VI, II-VI, and III-V colloidal quantum dots. *Chemical Communications* **2017**, *53*, 388–391.

(10) Zhang, J.; Zhang, H.; Cao, W.; Pang, Z.; Li, J.; Shu, Y.; Zhu, C.; Kong, X.; Wang, L.; Peng, X. Identification of Facet-Dependent Coordination Structures of Carboxylate Ligands on CdSe Nanocrystals. *Journal of the American Chemical Society* **2019**, *141*, 15675–15683.

(11) Guillemeney, L.; Lermusiaux, L.; Landaburu, G.; Wagnon, B.; Abécassis, B. Curvature and self-assembly of semi-conducting nanoplatelets. *Communications Chemistry 2022 5:1* **2022**, *5*, 1–11.

(12) Kim, D.; Lee, D. C. Surface Ligands as Permeation Barrier in the Growth and Assembly of Anisotropic Semiconductor Nanocrystals. *Journal of Physical Chemistry Letters* **2020**, *11*, 2647–2657.

(13) Balankura, T.; Yan, T.; Jahanmahin, O.; Narukatpichai, J.; Ng, A.; Fichthorn, K. A.; Fichthorn, K. A. Oriented attachment mechanism of triangular Ag nanoplates: a molecular dynamics study. *Nanoscale Advances* **2020**, *2*, 2265–2270.

(14) Liu, Z.; Zhou, H.; Lim, Y. S.; Song, J.-H.; Piao, L.; Kim, S.-H. Synthesis of Silver Nanoplates by Two-Dimensional Oriented Attachment. *Langmuir* **2012**, *28*, 9244–9249.

(15) Kim, M. H.; Yoon, D. K.; Im, S. H. Growth pathways of silver nanoplates in kinetically controlled synthesis: bimodal versus unimodal growth. *RSC Advances* **2015**, *5*, 14266–14272.

(16) Momper, R.; Zhang, H.; Chen, S.; Halim, H.; Johannes, E.; Yordanov, S.; Braga, D.; Blülle, B.; Doblas, D.; Kraus, T.; Bonn, M.; Wang, H. I.; Riedinger, A. Kinetic Control over Self-Assembly of Semiconductor Nanoplatelets. *Nano Letters* **2020**, *20*, 4102–4110.

(17) Jana, S.; Davidson, P.; Abécassis, B. CdSe Nanoplatelets: Living Polymers. *Angewandte Chemie* **2016**, *128*, 9517–9520.

(18) Midya, J.; Rubinstein, M.; Kumar, S. K.; Nikoubashman, A. Structure of polymer-grafted nanoparticle melts. *ACS Nano* **2020**, *14*, 15505–15516.

(19) Girard, M.; Ehlen, A.; Shakya, A.; Bereau, T.; de la Cruz, M. O. Hoobas: A highly object-oriented builder for molecular dynamics. *Computational Materials Science* **2019**, *167*, 25–33.

(20) Anderson, J. A.; Lorenz, C. D.; Travesset, A. General purpose molecular dynamics simulations fully implemented on graphics processing units. *Journal of Computational Physics* **2008**, *227*, 5342–5359.

(21) Glaser, J.; Nguyen, T. D.; Anderson, J. A.; Lui, P.; Spiga, F.; Millan, J. A.; Morse, D. C.; Glotzer, S. C. Strong scaling of general-purpose molecular dynamics simulations on GPUs. *Computer Physics Communications* **2015**, *192*, 97–107.

(22) Anderson, J. A.; Glaser, J.; Glotzer, S. C. HOOMD-blue: A Python package for high-performance molecular dynamics and hard particle Monte Carlo simulations. *Computational Materials Science* **2020**, *173*, 109363.

(23) Martyna, G. J.; Tobias, D. J.; Klein, M. L. Constant pressure molecular dynamics algorithms. *The Journal of Chemical Physics* **1994**, *101*, 4177.

(24) Tuckerman, M. E.; Alejandre, J.; López-Rendón, R.; Jochim, A. L.; Martyna, G. J. A Liouville-operator derived measure-preserving integrator for molecular dynamics simulations in the isothermal–isobaric ensemble. *Journal of Physics A: Mathematical and General* **2006**, *39*, 5629.

(25) Yu, T. Q.; Alejandre, J.; López-Rendón, R.; Martyna, G. J.; Tuckerman, M. E. Measure-preserving integrators for molecular dynamics in the isothermal–isobaric ensemble derived from the Liouville operator. *Chemical Physics* **2010**, *370*, 294–305.

(26) Stukowski, A. Visualization and analysis of atomistic simulation data with OVITO–the Open Visualization Tool. *Modelling Simul. Mater. Sci. Eng.* **2010**, *18*, 015012.

(27) Kästner, J. Umbrella sampling. *Wiley Interdisciplinary Reviews: Computational Molecular Science* **2011**, *1*, 932–942.

(28) Kumar, S.; Rosenberg, J. M.; Bouzida, D.; Swendsen, R. H.; Kollman, P. A. THE weighted histogram analysis method for free-energy calculations on biomolecules. I. The method. *Journal of Computational Chemistry* **1992**, *13*, 1011–1021.

(29) Souaille, M.; Roux, B. Extension to the weighted histogram analysis method: combining

umbrella sampling with free energy calculations. *Computer Physics Communications* **2001**, *135*, 40–57.

(30) Bauer, D. WHAM - An efficient weighted histogram analysis implementation written in Rust. (v1.1.3) [Software]. Zenodo. 2021; `https://doi.org/10.5281/zenodo.5121486`.

(31) Nath, S. K.; Escobedo, F. A.; Pablo, J. J. D. On the simulation of vapor–liquid equilibria for alkanes. *The Journal of Chemical Physics* **1998**, *108*, 9905.

(32) Widmer-Cooper, A.; Geissler, P. Orientational ordering of passivating ligands on CdS nanorods in solution generates strong rod-rod interactions. *Nano Letters* **2014**, *14*, 57–65.

(33) Phillips, C. L.; Anderson, J. A.; Glotzer, S. C. Pseudo-random number generation for Brownian Dynamics and Dissipative Particle Dynamics simulations on GPU devices. *Journal of Computational Physics* **2011**, *230*, 7191–7201.

(34) Petersen, N.; Girard, M.; Riedinger, A.; Valsson, O. Supporting dataset for the publication "The Crucial Role of Solvation Forces in the Steric Stabilization of Nanoplatelets" (1.2) [Data set]. 2022; `https://doi.org/10.5281/zenodo.7385599`.
